# The influence of vocal expertise on the perception of microrhythm in song and speech

**DOI:** 10.3758/s13414-025-03057-y

**Published:** 2025-04-25

**Authors:** Justin London, Thea S. Paulsrud, Anne Danielsen

**Affiliations:** 1 RITMO Center for Interdisciplinary Studies of Rhythm, Time, and Motion, University of Oslo, Oslo, Norway; 2 Carleton College, Northfield, MN USA; 3 Department of Musicology, University of Oslo, Oslo, Norway

**Keywords:** P-center, Timing perception, Expertise, Musical genre, Vocal performance, Speech perception, Music cognition, Sound recognition, Psychoacoustics, Temporal processing

## Abstract

Musical expertise affects the perception of the temporal location of a musical sound, often to a significant degree. In a study involving jazz musicians, electronic dance music (EDM) music producers, and Norwegian folk musicians, Danielsen et al. *Attention, Perception & Psychophysics*, *84*, 599–615, (2022) found significant between-group differences regarding the mean location as well as the variability around that mean for a set of control and genre-specific musical sounds. The present study extends these findings to singers who are experts in jazz versus classical music genres. In two experiments participants were presented with sung (Exp. 1) and sung and spoken (Exp. 2) syllables. In both the task was to synchronize either taps or a click track with a looped target sound. In both experiments, we found that classical participants tended to place the mean location later (relative to the acoustic onset) than jazz participants, and likewise had greater variability. Interestingly, and contra to our hypothesis, this between-group difference persisted even when the stimuli were spoken rather than sung. The current study gives further insights into how musical expertise impacts the low-level processing of musical sounds and provides a window into the interaction between top-down and bottom-up aspects of music and auditory perception more generally. In addition, it provides insight into how musicians approach musical and quasi-musical tasks, as well as the way they perceive the acoustic aspects of sound both as a musical object in its own right as well as a cue for perception-action coupling with their fellow musicians.

**Supplementary information:**

The online version contains supplementary material available at 10.3758/s13414-025-03057-y.

## Introduction

Our musical enculturation affects both how we hear and how we perform music. Musicians are better at synchronizing with familiar versus unfamiliar rhythmic patterns (Polak et al., 2018), non-musician listeners are better at detecting rhythmic glitches in familiar versus unfamiliar musical styles (Hannon et al., 2012), and people all over the world gravitate toward familiar rhythmic prototypes when asked to reproduce a random rhythmic sequence (Jacoby et al., 2024). Differences in rhythm perception and production between musicians and non-musicians are well documented, with musicians being better at both perceptual tasks (judgments of duration, synchrony, and whether a note is early or late relative to an established endogenous pulse) as well as behavioral tasks, such as maintaining a steady beat or tapping in synchrony with a pacing stimulus (for a summary of this literature, see Danielsen et al., 2022).

What has received less attention, however, are differences in the perception and cognition of sound and rhythm among different *sub-cultures* within broader musical traditions, especially as may be evident in the perception and performance by experts in particular musical genres. Expert musicians, whether they perform jazz, rock, classical, folk, or another musical style (to name a few in contemporary Western musical practice) develop skills that are specific to both their instrument and their chosen musical genre. For example, violinists and singers have keen senses of intonation and timbral color, while pianists develop complex forms of bimanual coordination when they play both melody and accompaniment (Jäncke et al., 2000; Nikjeh et al., 2009; Tervaniemi et al., 2005). In addition, all expert musicians are able to play with great rhythmic subtlety and nuance, as well as precisely coordinate their playing or singing with their fellow musicians. But as is also well known, musicians who are experts in one musical style may not exhibit the same skill when asked to play in other musical styles, often to their frustration.

This actualizes the question of the extent to which musical expertise, i.e., learned top-down processes, might impact the low-level, bottom-up processing of musical sounds. While musical training in general has been the topic of many studies, the extent to which training – especially high levels of training – in a particular musical style has an effect is an under-investigated topic in research into music perception and production. When given a generic musical or quasi-musical auditory task, no differences amongst musicians are usually found, for example, detecting the alignment of a metronome with a musical excerpt (Matthews et al., 2016), or pitch discrimination for instrumentalists versus vocalists (Nikjeh et al., 2009). However, when presented with tasks or tests that engage their instrumental and/or generic expertise, differences emerge. Margulis et al. (2009) noted differences in brain activity between flutists and violinists when listening to similar music played on their own versus the others'instruments, and Pantev et al. (2001) found enhanced sensitivity for violinists and trumpeters when presented with tones produced by the instruments with which they were most familiar.

Research has also found differences not only between musicians trained on different instruments, but also between musicians trained in different styles of music. Using a mismatch-negativity paradigm (MMN), Vuust et al. (2012) found differences in pitch and timbre processing between jazz, classical, rock/pop, and non-musicians when listening to music from different styles, and Hansen et al. (2016) found that jazz and classical musicians had different structural expectations when listening to jazz saxophone solos. Kliuchko et al. (2019), employing the same paradigm, found generally larger MMN amplitudes in response to deviants typical of jazz in jazz musicians, but not in classical musicians. Likewise, Tervaniemi et al. (2016) found differences in MMN responses amongst jazz, classical, and rock musicians when presented with deviants in tuning, timbre, rhythm, melody transposition, and melody contour. Using a non-auditory paradigm where classical and jazz pianists were exposed to images of incongruent harmonies and incorrect fingering, Bianco et al. (2018) found neural differences between musicians from the two genres reflecting differential calibration of hierarchical planning processes. Behaviorally this manifested in structure flexibility in jazz pianists and fine movement accuracy in classical pianists during the execution of the same task. Collectively, these MMN studies, which inherently involve cortical structures, provide evidence of top-down-driven differences in brain responses when musicians with different backgrounds listen to the exact same sound.

All of these studies make clear that musical expertise involves both instrument- and style-specific components. An obvious reason for this lack of transfer is that the perceptual-motor skills that are involved in expert musical performance are highly domain-specific: musicians spend thousands of hours learning to perform the melodies and rhythms of a very particular music style, whether jazz, classical, or bluegrass. This embodied procedural knowledge explains differences in performance/motor behavior, that is, in what expert musicians are able to do (Manning & Schutz, 2016; Matthews et al., 2016; Polak et al., 2018; Zatorre et al., 2007).

Danielsen et al. (2022) found evidence that instrumentalists and music producers who are experts in different styles do not hear the musical sounds the same way. In that study, expert participants were recruited from three musical genres: jazz, Scandinavian traditional fiddle music, and a subset of popular music styles: electronic dance music (EDM), electro-pop, trap, and hip-hop. These experts were presented with looped musical stimuli using sounds that were “native” to each genre, along with a set of artificial sounds used as controls. On separate trials they were asked to either synchronize a click track or tap along using a set of clave sticks with the repeating target stimulus. Each stimulus category had a balanced 2 × 2 design of the acoustic factors of Attack/Rise Time (soft/slow vs. sharp/fast) and Duration (short vs. long). The dependent measures were the average location of clicks or taps relative to the acoustic onset of each target sound/stimulus, as well as the variability of click or tap locations within each group of participants.

These stimuli and tasks were used to probe the participants’ senses of the *perceptual center*, or P-center, of each stimulus. The term *P-center* was first used to describe the perceived temporal location of speech sounds in phonetics (Morton et al., 1976), especially when those sounds are presented in a repeated, cyclical fashion. P-centers are not simply the perceived moment of a sound’s occurrence once some amplitude threshold has been reached (i.e., not mere sonic detectability), but can occur well after the initial sound onset, and are in speech related to our perception of the articulatory actions involved in producing the sound (Scott, 1998; Tuller & Fowler, 1980). The term was then later applied to the perception of musical sounds (Gordon, 1987; Villing, 2010; Vos & Rasch, 1981). In a metrical context, that is, in music with a regular beat or pulse, a P-center is analogous to the location of the beat relative to the acoustic onset of a tone. A constellation of acoustic factors, in particular amplitude rise time and sound duration (Danielsen et al., 2019; Gordon, 1987; London et al., 2019; Villing, 2010; Vos & Rasch, 1981), as well as center frequency, and spectral composition/timbre (Danielsen et al., 2019; Hove et al., 2007) have been found to affect P-center perception in music. Moreover, P-centers are best regarded not as points in time after the onset of a sound, but rather as the probability distribution of a given sound’s perceived temporal location (Danielsen et al., 2019; Gordon, 1987; Wright, 2008), which Danielsen (2010, 2019) has characterized as the “beat bin.” P-centers/beat bins thus have both a peak (the mean location of taps or clicks synchronized with the sound) and a spread (the dispersion of those taps or clicks about the mean location).

**Fig. 1:**
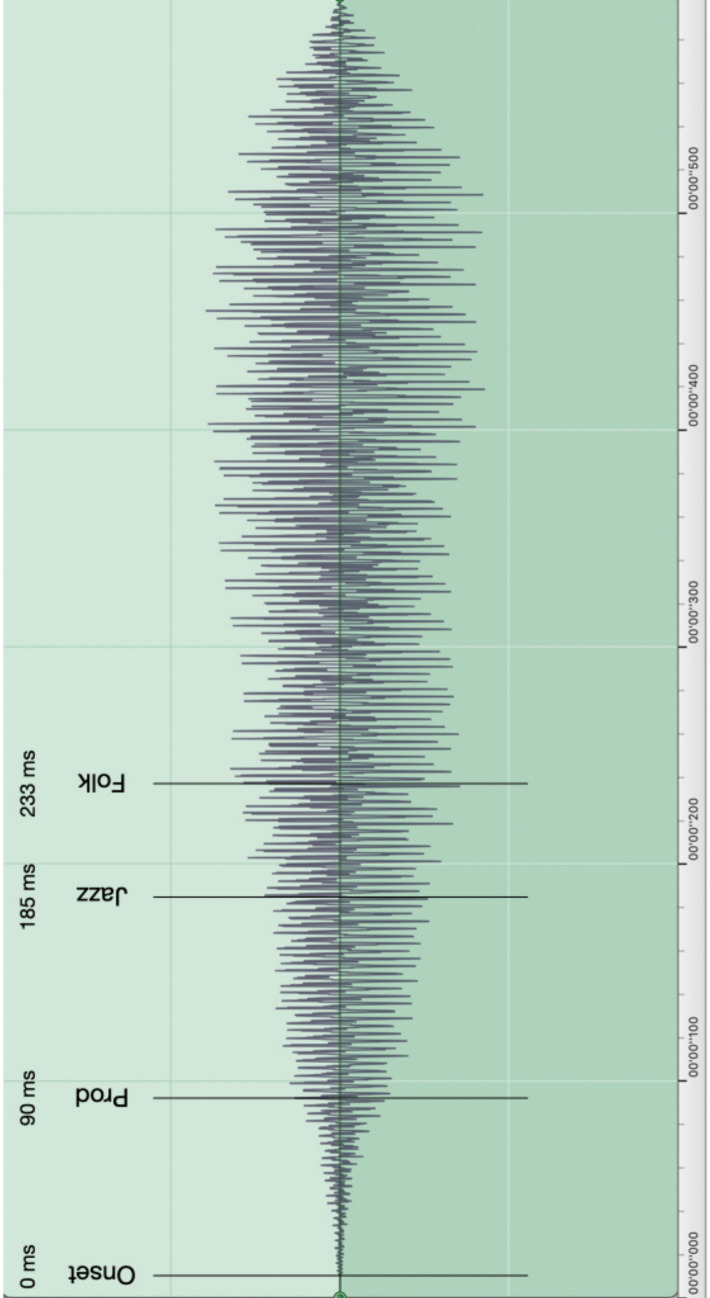
Waveform of the long fiddle sound with mean P-center locations in the click alignment task for each of the three participant groups in Danielsen et al. (2022)

If P-center perceptions are driven by acoustic factors alone, and the low-level perceptual mechanisms for detecting and encoding those factors, then there should be little difference amongst listeners, whether due to training or enculturation. However, Danielsen et al. (2022) found significant differences amongst their three expert participant groups: producers of computer-based popular music located the click or tap, on average, earlier than both the folk and jazz musicians, and, as expected based on their training, folk musicians had overall significantly wider beat bins (higher variability) than the producers. One stimulus in particular gave rise to a particularly striking contrast (see Fig. 1).

The long fiddle sound produced extreme differences as regards both P-center location and variability (i.e., beat bin width) among the three participant groups, with extremely “late” P-centers and wider beat bins for the folk and jazz musicians in comparison to the producers. Thus Danielsen et al. (2022) found that (a) genre expertise can effect low-level perceptions of sounds, as even the control sounds produced small but significant differences in P-center variability/beat bin width amongst the three groups, and (b) genre expertise can have a top-down influence on bottom-up processing in terms of activating genre-specific timing ideals, as evidenced by the striking P-center and variability results for the long fiddle sound typical of the Scandinavian fiddle music tradition.

The current experimental study probes whether the findings of Danielsen et al. (2022) are transferrable to other musician populations, specifically vocalists. One confound in our previous study was that we did not control for specific instruments within each of our different expert participant groups. Musical instruments function as cognitive extensions, allowing their performers to “offload” many aspects of musical sound production, which may include tuning, articulation, volume control, and timbre (Clark & Chalmers, 1998; Clark, 2008; Magnusson, 2009; Polanyi, 1962). Thus, some of the between-group differences we observed may have been due to the clustering of specific instruments within participant groups. By contrast, for vocalists any top-down perceptual effects due to expert training in a particular musical style are uninfluenced by the acoustical and physical limitations regarding the sounds the instrument can produce and the ergonomic factors involved in playing it. While sharing important aspects of basic training in breathing technique, articulation, use of different vocal registers, etc., there are considerable differences in technique and aesthetic ideals between vocalists trained in different musical styles. A major division is between classical vocal training and a range of different popular styles that in North American voice pedagogy are often labeled “Contemporary Commercial Music” or CCM (Bartlett & Naismith, 2020). Characteristics separating the two traditions are voice quality, tone, and registration, as well as genre/style-specific features. For example, systematic differences in the use of vibrato between professional Western operatic singers and jazz vocalists have been identified (Manfredi et al., 2015). Several studies (Björkner, 2008; Schutte & Miller, 1993) have observed differences in subglottal pressure, voice source, and formant frequency characteristics involved in “belting,” a vocal technique commonly used in non-classical singing styles. The “belting” voice takes the chest register to higher pitches than is generally advised in classical singing, and the higher vocal tract (larynx) position and higher subglottal pressure make the sound quite different from the “classical” chest voice. Further distinctions in subglottal pressure have also been identified *within* the field of different forms of popular music singing styles (Borch & Sundberg, 2011; Guzman et al., 2015; Hallqvist et al., 2017). Belting stands in contrast to operatic bel canto singing, with “bel canto” understood here as the characteristic sound of a “classically” trained voice for opera and concert singers (Stark, 1999). Previous research has also documented that such stylistic differences among singers are immediately recognized by experts (Borch & Sundberg, 2011; Kayes & Welch, 2017).

Another significant difference between classical and the CCM vocal genres is their distinctly different ways of shaping the attack portion of vocal sounds. In bel canto singing style the onset of notes should be smooth and support an overall legato (i.e., connected) sense of melody that foregrounds melodic contour. In CCM vocal genres, on the other hand, speech-like qualities and a rhythmically punctuated, “percussive” approach are often preferred (Bartlett & Naismith, 2020; Danielsen, 2006; Robinson-Martin, 2009). As our previous research has shown, sharp (i.e., fast) attacks typical of percussive sounds engender high temporal precision, whereas soft (i.e., slow) attacks produce a larger temporal window for synchronizing with a virtual beat or another instrument (Danielsen, 2010; Danielsen et al., 2019, 2022, 2024; London et al., 2019). In contrast to classical singers, who are trained to avoid sharp onsets, jazz singers use both smooth and sharp attacks (Jacobsen & Danielsen, 2023). The latter is particularly important when engaging in a rhythmic dialogue with the accompanying instruments in the rhythm section of the band. Given these differences in aesthetic ideals, training, and approaches to the production of sound and rhythm, might these habits of vocal production affect how singers trained in different musical styles *perceive* musical sounds?

The current experimental study continues and extends the method used in Danielsen et al. (2022). In the first experiment (Experiment 1), we used a set of vocal stimuli produced by jazz and classical singers, as well as a subset of the control sounds used in the previous study, and asked expert jazz^1^ and classical singers to perform the same click alignment and tapping tasks. The same dependent measures (click or tap mean location and their corresponding mean variabilities) were obtained. In the current study all the participants were trained female vocalists educated in music conservatories in which jazz and classical singers receive distinctly different forms of vocal training.

As with our previous study, we expect that there will be differences between the two participant groups, both in general and specifically with respect to a participant’s relation to a given sound (i.e., if the stimulus is a sound that is familiar/from their own genre, vs. sounds that are not). In addition, we expect to replicate the effect of attack rise time (*Note*: duration was not manipulated in the present study, as the differences between subjects arise most clearly in stimuli with longer durations). As noted above, jazz singers are trained to accentuate the attack of a tone, given the rhythmic style of much jazz performance, while classical singers are trained to have a legato singing style which entails a softer attack. From our previous work, we know that sounds with softer attacks give rise to later P-centers and greater P-center variability. Hence hypotheses for Experiment 1 are as follows:

Responses to Control stimuli:

- Control stimuli with soft attacks will have later P-centers and greater variability than Control stimuli with sharp attacks, as per Danielsen et al. (2022).
- When presented with Control stimuli, jazz and classical singer-participants will have similar responses for P-center location and variability.

Responses to Vocal stimuli:

- P-center location: Jazz singer-participants will have earlier P-center locations than classical singers.
- P-center variability: Classical singer-participants will have greater P-center variability than jazz singers.

Effects of Musical style within Vocal stimuli:

- Vocal stimuli produced by classical singers, which have a longer attack and distinct vibrato onset within the sound in comparison to vocal stimuli produced by jazz singers, will give rise to both later P-centers and greater variability for all participants, in line with previous studies (Danielsen et al., 2019; Gordon, 1987; London et al., 2019; Villing, 2010; Vos & Rasch, 1981).

An exploratory aspect of the current study will be to examine the various affordances each stimulus offers for the location(s) of P-centers and whether such affordances vary between groups, as was found with the long fiddle sound given in Fig. 1.

From previous experiments, both ours and others, we know that musical expertise is highly context-specific, which gives rise to the differences between different groups of musical experts that we have observed. Thus, when a listening context invites a musician to make use of their expertise, they will do so. In ordinary conversation, however, we presume that this expertise is not relevant, and other factors (e.g., particular nuances of dialect, the effect of secondary languages, etc.) will be far more relevant. If we presume those factors are randomly distributed in our participant population, we then expect that when presented with spoken as opposed to musical stimuli, our expert participants will not exhibit the between-group differences that we have found for musical stimuli. In Experiment 2, we used a subset of the sung stimuli from Experiment 1 in combination with matched spoken syllables, and we asked a similar population of jazz and classical singers to perform the same click alignment task. The spoken syllables were produced by the same singers as in Experiment 1. To control for any carry-over effects (i.e., if participants recognized the particular voices of the classical and jazz singers who produced both the sung and spoken stimuli), we also included a set of spoken syllables produced by a female speaker without vocal training. Our hypotheses for Experiment 2 are:

- We would replicate the findings of Experiment 1 for the sung stimuli, specifically the between-group differences for P-center location and variability
- That for the spoken stimuli, however, we would not observe any between-group differences for P-center location and variability.

## Experiment 1

### Methods

#### Participants

As the stimuli were produced by female singers in each of the two genres, we recruited only female singers as participants for the experiment, as matching the vocal range and gender of participants and stimuli would give their expertise the greatest affordance (see Calvo-Merino et al., 2010). Participants were recruited from the Norwegian Academy of Music, Oslo, the Department of Musicology at the University of Oslo, the Department of Music at the Norwegian University of Science and Technology, Trondheim, and the Department of Music at the University of Bergen. Initially, experts in folk music were recruited as a separate participant group, but due to difficulty in recruiting a large enough group of participants, and given that preliminary results indicated that the folk singers’ responses by and large duplicated those of jazz singers, the final set of participants consisted of 18 classical singers and 18 jazz singers (age range 21–60 years; average age 27.6 years). Written informed consent was obtained and each participant received a gift card (approx. value 20 USD) for their participation in the experiment.

#### Stimuli

The stimuli consisted of looped/repeated presentations of the target sounds. The four Control sounds were taken from our previous experiment. The two artificial sounds were 400 ms in duration with a center frequency of 100 Hz, and differed only in their attack rise time (sharp = 3 ms, soft = 50 ms). They were generated in Max 7, using white noise and bandpass-filters with a Q-factor of 10. The amplitude of the sound files was scaled linearly from 0 (beginning of file) to 1 (at the indicated rise time), immediately followed by a linear decay to silence at the end of each sound file. The click sound was 1 ms in duration with a spectral centroid of 3,531.5 Hz, and the bowed fiddle sound (Fig. 1) was on the pitch D1 (349.2 Hz) and had a duration of 580 ms.

To create natural-sounding vocal stimuli, and to make sure that all stimuli were clearly identifiable as to the musical genre of each singer, stimuli were created and selected in the following fashion. First, six experienced female vocalists (three from each genre) were recruited to produce the stimulus sounds. All three were mezzo-soprano or alto vocal ranges, though some of the classical singers could sing in higher ranges as well (i.e., lyric sopranos). All of the singers were native speakers of Norwegian, save for one classical singer whose native language was Swedish.

All singers were asked to sing two different vowel sounds (“a, e”; in IPA “ɐ, ǝ,” i.e., central vowels in open-mid and close-mid position), either without any initial consonant (sung on the pitch Eb4), or with an initial “m” (bilabial nasal consonant), sung on G4 (pitch differences were included for variety; both fall in the same vocal range). Stimuli were recorded at the University of Oslo Department of Musicology’s sound studio, with ProTools as the recording software and with a Neumann U87 Ai microphone.

Since the stimuli were to be presented in looped fashion, the singers recorded each sound in a metrical context to produce naturally sounding repeated stimuli. At the start of each recording singers heard four metronome clicks at the target BPM (beats per minute) rate. Singers then waited four beats (mentally maintaining the initial BPM rate), and then sang; they continued to maintain the internal beat, and then sang again on two more successive “downbeats.” The stimulus target tempo was initially 80 BPM, so singers were given clicks at a rate of 82 BPM, slightly faster to ensure that each tone would be completed within the span of a single beat. From each take, one sound was selected as a candidate for use in the main experiment.

Having created a large set of potential stimuli (each target sound sung by three different singers), all vocal stimuli were presented to a group of seven raters, all of whom were familiar with both vocal styles. Each rater was asked to (a) identify the genre background of the singer, and (b) give a confidence rating (5-point scale). For each stimulus category, the stimulus that was chosen for use in the main experiment was correctly identified by all raters and was given the highest confidence rating. This ensured that each stimulus would be regarded as a vocalization characteristic of each genre. The final set of sounds used in the experiment are given in Table 1.^2^

**Table 1:** Sounds used in Experiment 1

Stimulus genre	“Sharp attack”		“Soft attack”	
Classical vocal stimuli	“A”	“E”	“Ma”	“Me”
Jazz vocal stimuli	“A”	“E”	“Ma”	“Me”
Control stimuli	Click	Sharp noise	Fiddle	Soft noise

#### Procedure, apparatus, and setup

During the click alignment (“CLICK”) trials the participants aligned a click track with a looped presentation of the target stimulus. Both the click track and stimuli were looped at an 800-ms interval (tempo = 75 BPM). Note this is slower than our initial target tempo of 80 BPM, as described above. Since many of the singers extended their tones slightly beyond the duration of the beat, in order to avoid extensive editing that might introduce unwanted artifacts, stimuli were presented at 75 BPM. The tail of two of the stimuli (classical “Ma” and “Me”) were given a slight fade-out to avoid clipping at the sound offset.

The click and target loops started with a random alignment offset of ≈100–200 ms. In each trial, participants manipulated the timing of the click track by moving an on-screen cursor using the mouse and/or arrow keys (one key click = 1 ms). Participants were also able to adjust the volume of the click track. When satisfied that the target stimulus was synchronized with the click track, participants moved to the next trial. Following two practice trials, participants heard each target stimulus three times for a total of 39 trials. The order of stimulus presentation was semi-random, constrained so that participants never heard the same stimulus on successive trials. There was no time limit for each trial in the click alignment task, and on average the participants used 38 s per trial. The overall time for each participant to perform all CLICK trials varied from 10 to 68 min.

Participants completed the CLICK task using a MacBook Pro computer (3.1 Ghz Intel core i7, OSX 10.11.16), listening via Beyer Dynamic DT 770 Pro headphones at a comfortable intensity that could be further adjusted by the participant. Stimuli were presented using a custom-made patch written in Max 8.2.2 (http://www.cycling74.com), which also recorded participants’ responses, in total 1,296 click locations (12 stimuli × 3 trials × 36 participants). P-centers are reported in milliseconds relative to the physical onset of each stimulus. Twenty-four trials were excluded because the click occurred more than 80 ms before the onset of the sound. The standard deviations of the three trials for each stimulus for each participant were also calculated. The grand averages of participants’ P-centers and standard deviations were used as measures of the overall CLICK P-center location and variability for each stimulus.

In the tapping trials (“TAP”), the task was to tap along using a pair of clave sticks in synchrony with the target stimulus, again looped at an 800-ms interval. There was one loop for each target stimulus which repeated for 20 s. The 12 loops were presented in random order. Participants were given two practice trials to gain familiarity with the clave sticks as well as with the task at hand. Participants took 5–10 min to finish the tapping trials. In the TAP task participants used acoustically transparent headphones (Koss PortaPro) which allowed them to clearly hear their tapping during those trials. To eliminate timing latencies during the Tapping task, the stimulus was split and routed both to participants’ headphones and to a mono recording channel on an audio interface (RME Babyface Pro or RME Fireface UCX); tapping data were recorded on another mono channel using a Shure SM57 unidirectional microphone.

A MATLAB (version R2022a) script was used to identify tap onsets, as the time point where the rectified tapping audio waveform first exceeded a predefined threshold close to the noise floor. For each registered tap, the time difference between its detected onset and the first zero crossing of the closest stimulus sound was calculated. Data were collected from 16 consecutive taps, beginning with the third tap of each trial. Tap/Stimulus onset asynchronies were averaged to calculate the TAP P-center for each stimulus per participant. One jazz participant had missing data for the four Control sounds. One series by one classical participant was excluded due to offbeat (antiphase) tapping. Of the remaining 427 data series (35 participants × 12 stimulus sounds + 1 participant × 8 sounds – 1 excluded data series), four series had fewer than 16 registered taps; in those cases all registered consecutive taps from the third tap were used, that is, 9, 13, 15, and 15 taps, respectively.

The standard deviation of the tap/stimulus asynchronies in each series (i.e., one per stimulus per participant) was also calculated. The grand averages of participants’ P-centers and standard deviations were used as measures of the TAP P-center location and TAP variability for each stimulus. Participants completed the CLICK trials first, and then did a block with the TAP task. Between the experimental tasks, participants completed a background questionnaire. Participants were encouraged to proceed through the experiment at their own pace and to take breaks as needed. One of the experimenters waited nearby should any questions/problems arise.

#### Statistical analysis

**Fig. 2:**
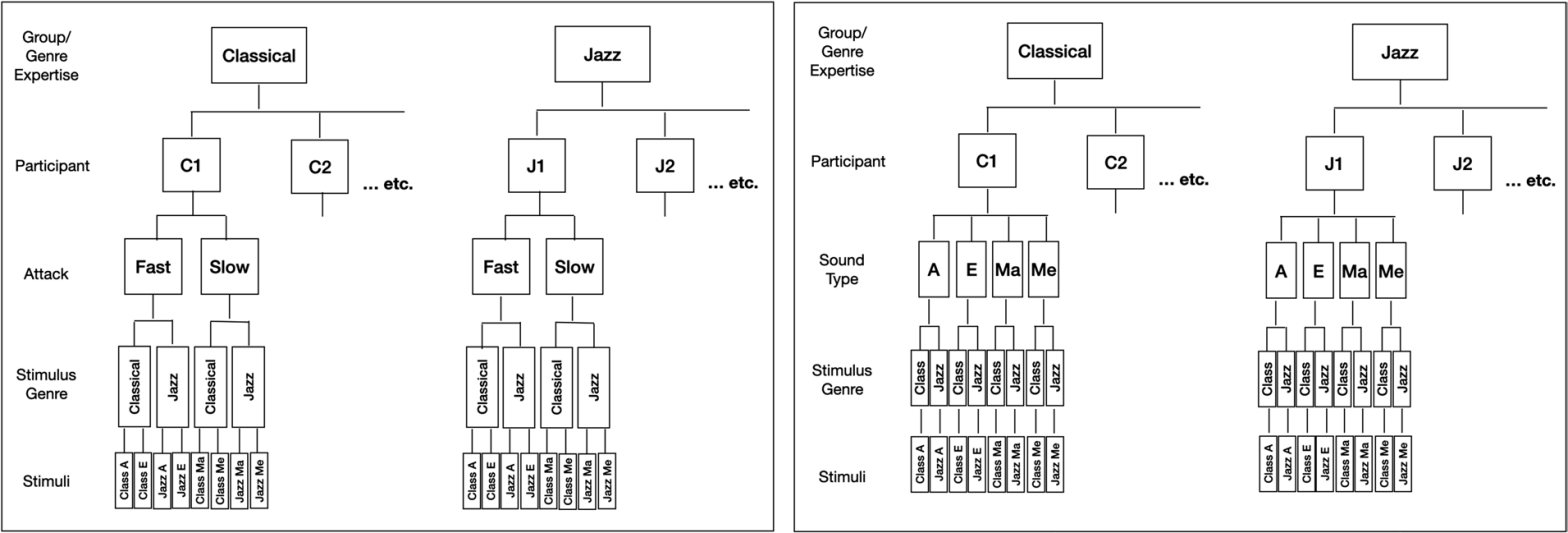
Planned (**left**) and exploratory (**right**) multilevel models for analysis of vocal data in Experiment 1

We analyzed vocal and control data separately. Responses to the control stimuli were analyzed using Linear Mixed Models with Group as between-subjects, Attack as within-subjects fixed effects, and Participant as random intercept. The responses to the vocal stimuli were analyzed using two different Multilevel Linear Mixed Models (see Fig. 2). The first (planned) model had Stimulus Genre (classical, jazz) nested in Attack (sharp, soft) as the within-subjects fixed effect, while the second exploratory model had Stimulus Genre nested in Sound Type (A, E, Ma, Me). Both had Group (classical, jazz) as the between-subjects effect and Participant as Random Intercept. P-center (e.g., click or tap location relative to stimulus onset) and variability (average click or tap location for each stimulus per participant) were the dependent variables in all models. Model fit was assessed by comparing AIC scores for full models to a null model with Intercept and Random Intercept (Participant), only. The analyses were performed using Satterthwaite approximations (Satterthwaite, 1946), restricted maximum likelihood estimation (REML), and a variance components covariance matrix. Residuals in all models were normally distributed. Follow-up paired-samples t-tests of group differences were then performed for each stimulus category (classical, jazz, and control), separately, plus the two vocal stimulus categories together. All tests were run with Bonferroni correction for multiple comparisons. Click alignment and tapping data were analyzed separately. To examine whether some of the stimuli afforded several P-centers (i.e., produced more than one peak of responses), we also examined the distributions of click locations in response to each stimulus separately in box-plots and histograms. All analyses were performed in SPSS 29.

### Results

We begin with descriptive statistics of the mean location and variability (per stimulus) for click alignment and tapping trials, including significant group differences for the stimulus categories (see Table 2). We then highlight the principal results of our linear mixed model analysis. (A complete account of the results of the linear mixed models and paired-samples t-tests is provided in Tables A1, A2, A3, A4 and A5 in the Appendix, Online Supplemental Material). We then zoom in on the distribution of responses for each stimulus sound separately.

**Table 2:** Summary statistics for P-center location and variability in milliseconds for all stimulus sounds and categories in Experiment 1, including CLICK and TAP tasks. Means for CLICK data are reported as mean of means for each participant’s three trials of each stimulus

	CLICK data						TAP data					
Stimulus/Group	P-center mean			Variability			P-center mean			Variability		
	Class.	Jazz	Total	Class.	Jazz	Total	Class.	Jazz	Total	Class.	Jazz	Total
Classical A	73	88	81	68	61	64	58	49	53	24	19	22
Classical E	51	29	40	50	29	40	31	32	32	23	19	21
Classical Ma	175	157	166	28	32	30	168	165	167	24	17	21
Classical Me	213	210	211	33	23	28	220	213	217	23	17	20
Avg. Classical stimuli	129	120	125	44	36	40	119	115	117	**24**	**18**	21
Jazz A	106	40	73	54	45	50	60	17	38	19	16	18
Jazz E	40	27	34	35	26	31	15	22	18	19	16	18
Jazz Ma	60	43	52	37	25	31	40	36	38	20	18	19
Jazz Me	55	43	49	36	31	33	25	33	29	21	16	18
Avg. Jazz stimuli	**66**	**39**	52	40	31	35	35	27	31	**20**	**17**	18
Avg. Vocal stimuli	**97**	**80**	88	43	34	38	77	71	74	**22**	**17**	20
Click	− 2	− 2	− 2	12	4	8	− 14	− 3	− 9	31	22	27
Sharp noise	13	13	13	32	18	25	13	14	14	17	17	17
Soft noise	39	36	37	30	19	24	32	32	32	17	16	17
Fiddle	117	81	99	49	53	51	94	62	78	28	21	24
Avg. Control stimuli	41	32	37	27	23	25	31	26	29	**23**	**19**	21

Bold numbers = significant group differences for the stimulus category at *p* <.05 (two-tailed)

#### Control stimuli

**Fig. 3:**
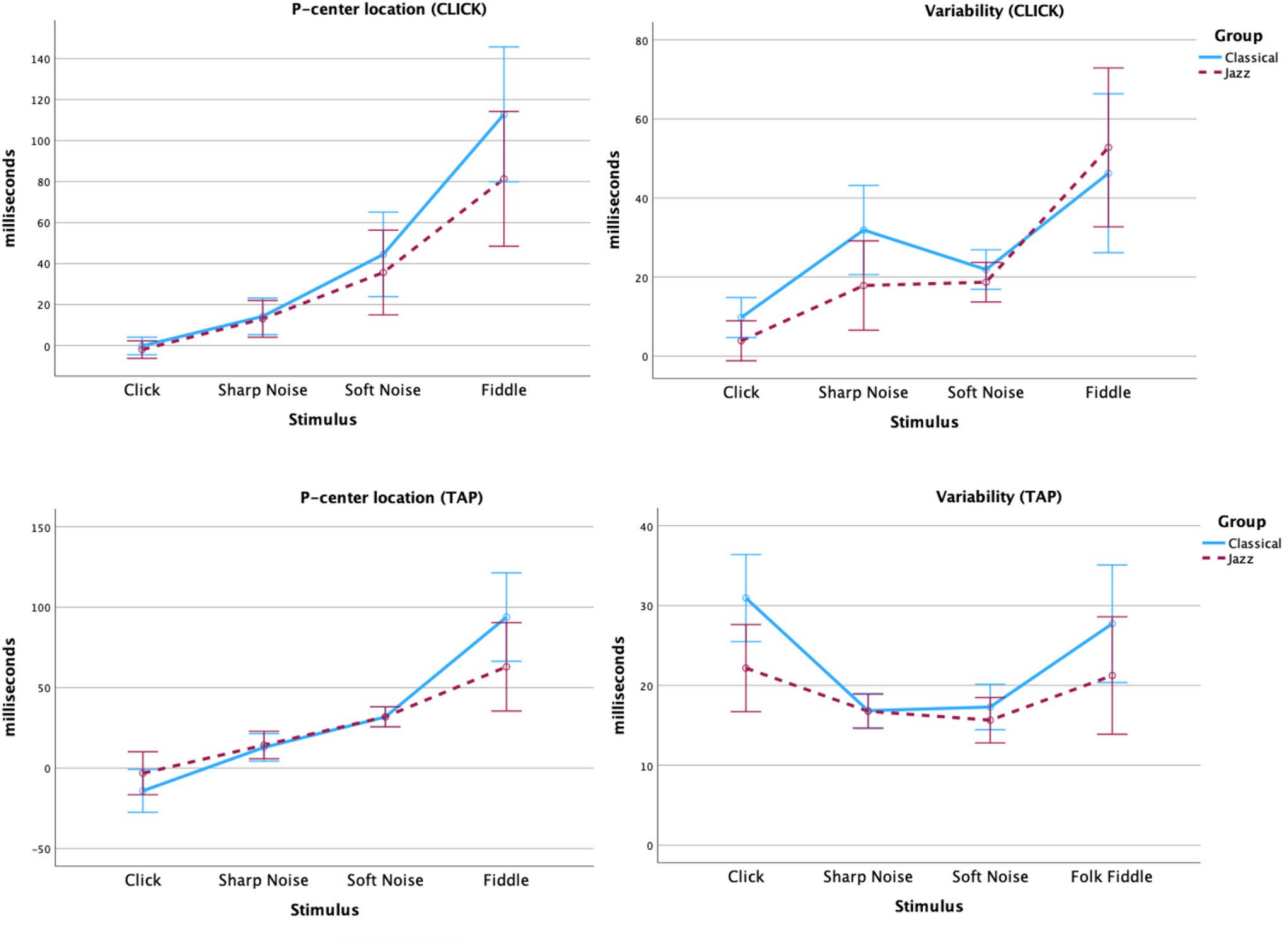
Plots of mean P-center location relative to stimulus onset (**left**) and variability (**right**) for each sound in the Control stimuli category in the CLICK (**top**) and TAP (**bottom**) task in Experiment 1, separated by Group/Participant genres. Error bars are 95% confidence intervals

Plots of results for each control sound in the click and tapping task are shown in Fig. 3.

*Click alignment task (CLICK):* There was no significant effect of Group, but significant effects of Attack on P-center location and variability (both at *p =*.001).

*Tapping task (TAP):* For P-center location there was no significant effect of Group, but there was a significant effect of Attack (*p =*.001). The model for the variability data did not converge. These data were thus analyzed by a mixed repeated-measures ANOVA with Group, Attack, and Stimulus as factors. The results show a main effect of Group (*p =*.018). (One missing data point was replaced by the grand average of all other participants for that stimulus.) The classical participant group had, on average, 4 ms higher variability than the jazz participants. There was no main effect of Attack or Stimulus, but there were significant interactions (Attack × Stimulus: *p* <.001, Attack × Stimulus × Group: *p =*.026). As can be seen in Fig. 3, bottom right panel, the click and fiddle sounds produced both higher variability and a greater difference between the two participant groups than both the sharp and the soft noise stimuli. For the click sound, all participants exhibited the typical negative mean asynchrony in the tapping task (Repp, 2005).

#### Vocal stimuli

**Fig. 4:**
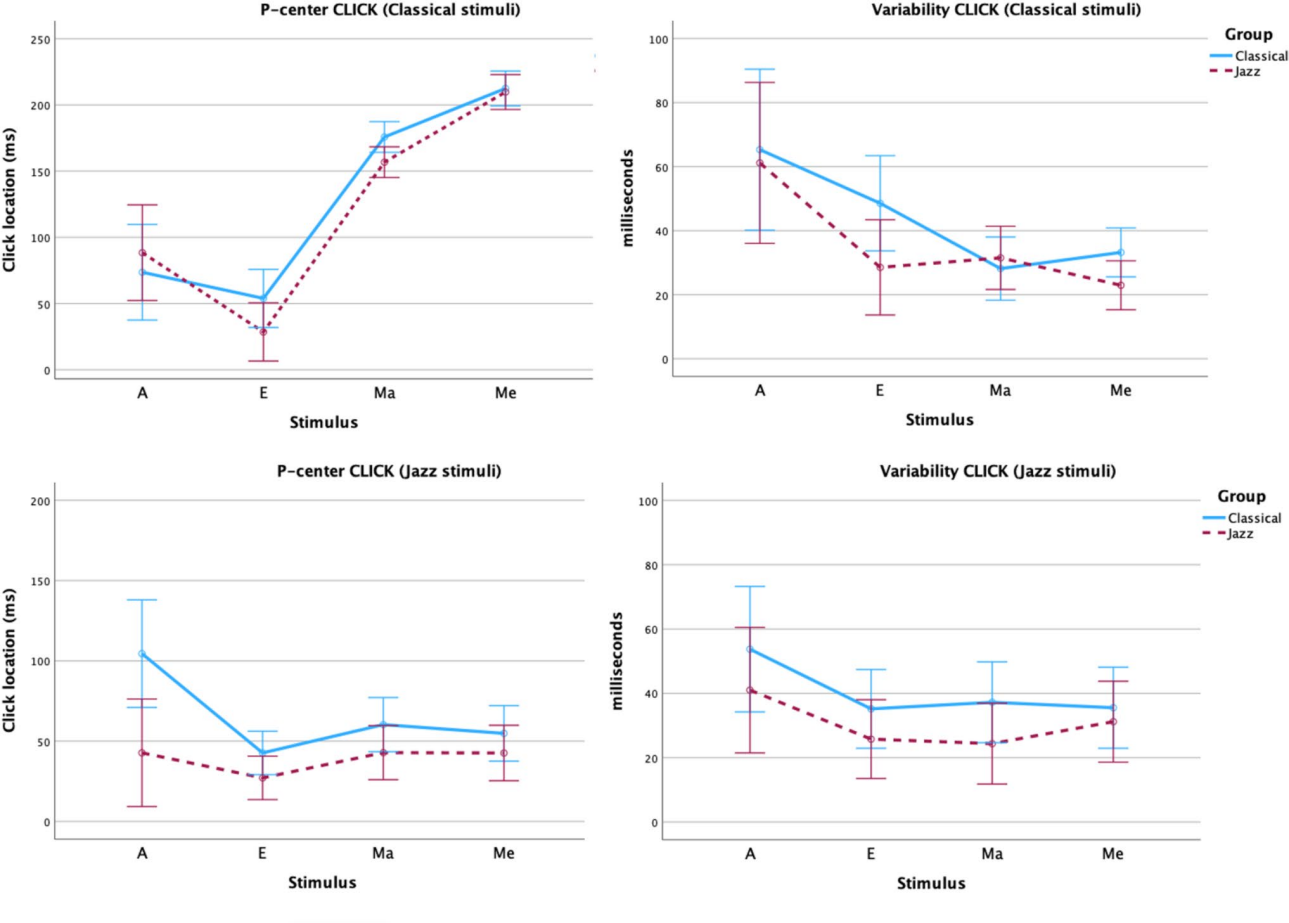
Plots of mean P-center location relative to stimulus onset (**left**) and variability (**right**) for each sound in the Classical Stimulus Genre (**top**), Jazz Stimulus Genre (**bottom**) in the click alignment task in Experiment 1, separated by participant genres. Error bars are 95% confidence intervals

**Fig. 5:**
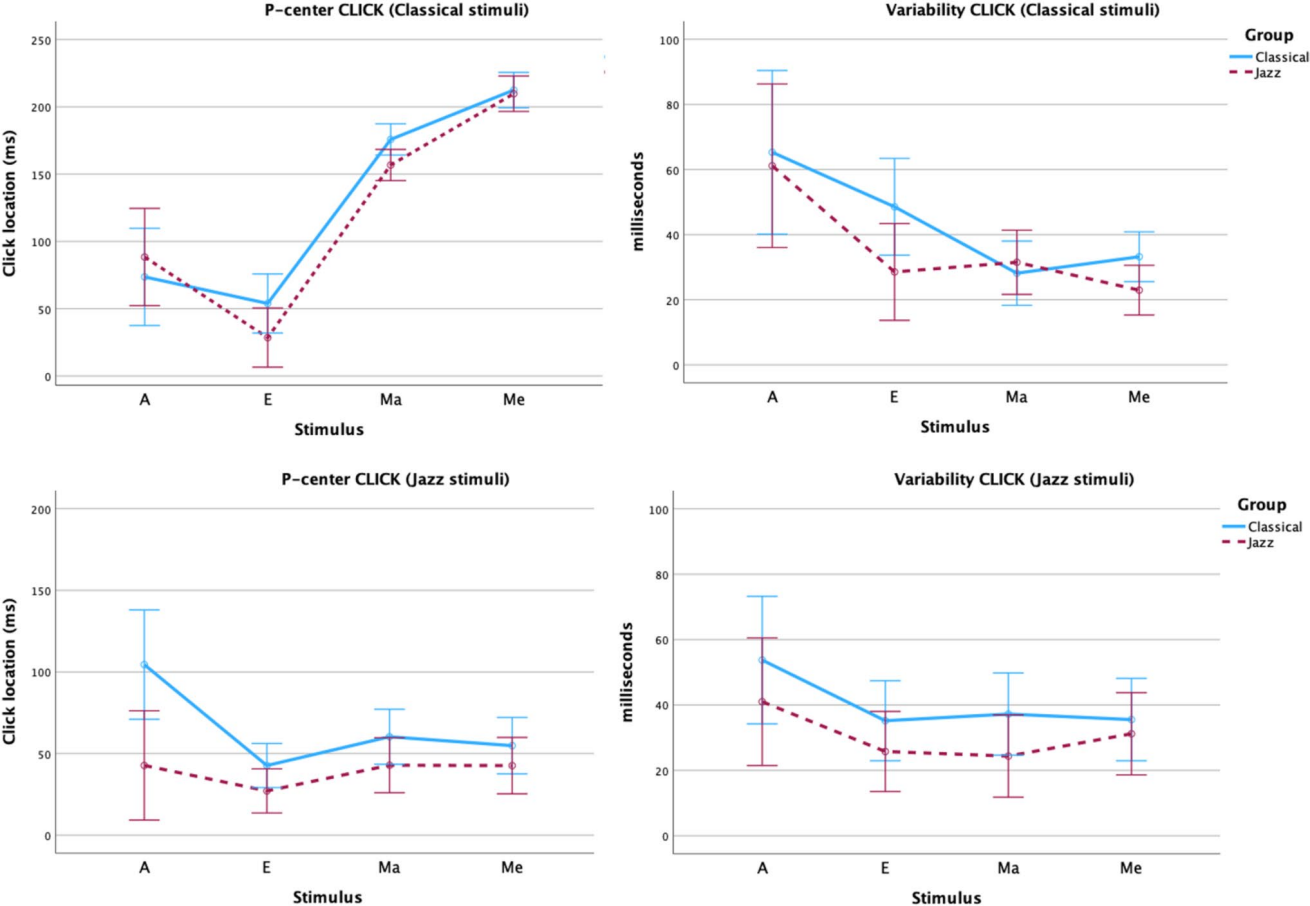
Plots of mean P-center location relative to stimulus onset (**left**) and variability (**right**) for each sound in the Classical Stimulus Genre (**top**), Jazz Stimulus Genre (**bottom**) in the tapping task in Experiment 1, separated by participant genres. Error bars are 95% confidence intervals

Plots of results for each sound in the click alignment and tapping tasks are shown in Figs. 4 and 5.

*Click alignment task:* The linear mixed model with Group and Stimulus Genre nested within Attack found a significant difference in P-center location (*p =*.031) between the two participant groups, as the classical participants had on average 17 ms later P-center locations (95% CI: 2–32) than the jazz participants. There was also a significant effect of the within-subjects factor Attack(Stimulus Genre) at *p <*.001 at the level Soft attack(Classical stimuli) with Sharp attack(Jazz stimuli) as the reference level. Changing the within-subjects factor from Attack(Stimulus Genre) to Sound Type(Stimulus Genre) gives a more fine-grained picture of how the different sounds contribute to the model (reference level = Jazz E), with significant effects of Jazz Me (*p <*.001), Jazz Ma (*p <*.001), Classical A (*p <*.001), Classical Me (*p <*.026), and Classical Ma (*p <*.001).

The linear mixed model with Group and Stimulus genre nested within Attack found no significant difference in P-center variability between the two participant groups, but there was a significant effect of the within-subjects factor Attack(Stimulus Genre) at the level Soft attack(Jazz stimuli) (*p =*.024) and Soft attack(Classical stimuli) (*p =*.029) with Sharp attack(Jazz stimuli) as the reference level. Again, changing the within-subjects factor from Attack(Stimulus Genre) to Sound Type(Stimulus Genre) shows a more fine-grained picture of how the different sounds contribute to the model (reference level = Jazz E), with significant effects at the levels Jazz Me (*p <*.007) and Jazz Ma (*p <*.001).

*Tapping task:* The linear mixed model with Group and Stimulus Genre nested within Attack found no significant difference in P-center location between the two participant groups but a significant effect of the within-subjects factor Attack(Stimulus Genre) at the levels Soft attack(Jazz stimuli) (*p =*.016) and Soft attack(Classical stimuli) (*p <*.001) with Sharp attack(Jazz stimuli) as the reference level. Changing the within-subjects factor from Attack(Stimulus Genre) to Sound Type(Stimulus Genre) shows that the levels Jazz Me (*p =*.009), Jazz Ma (*p <*.001), Classical A (*p <*.001), Classical Me (*p =*.009), and Classical Ma (*p <*.001) contribute significantly to the model (reference level = Jazz E).

The linear mixed model with Group and Stimulus Genre nested within Attack found a significant difference in P-center tapping variability (*p =*.011) between the two participant groups, such that the classical participants had, on average, 4 ms higher variability (95% CI: 1–8) than the jazz participants. There was also a significant effect of the within-subjects factor Attack(Stimulus Genre) at the levels Soft attack(Jazz stimuli) (*p =*.004), and Soft attack(Classical stimuli) (*p =*.032); reference level was Sharp attack(Jazz stimuli). Changing the within-subjects factor from Attack(Stimulus Genre) to Sound Type(Stimulus Genre) shows that the levels Jazz Me (*p =*.007) and Jazz Ma (*p <*.001) contributed significantly to the model (reference level = Jazz E).

#### Distribution of responses for each stimulus sound

**Fig. 6:**
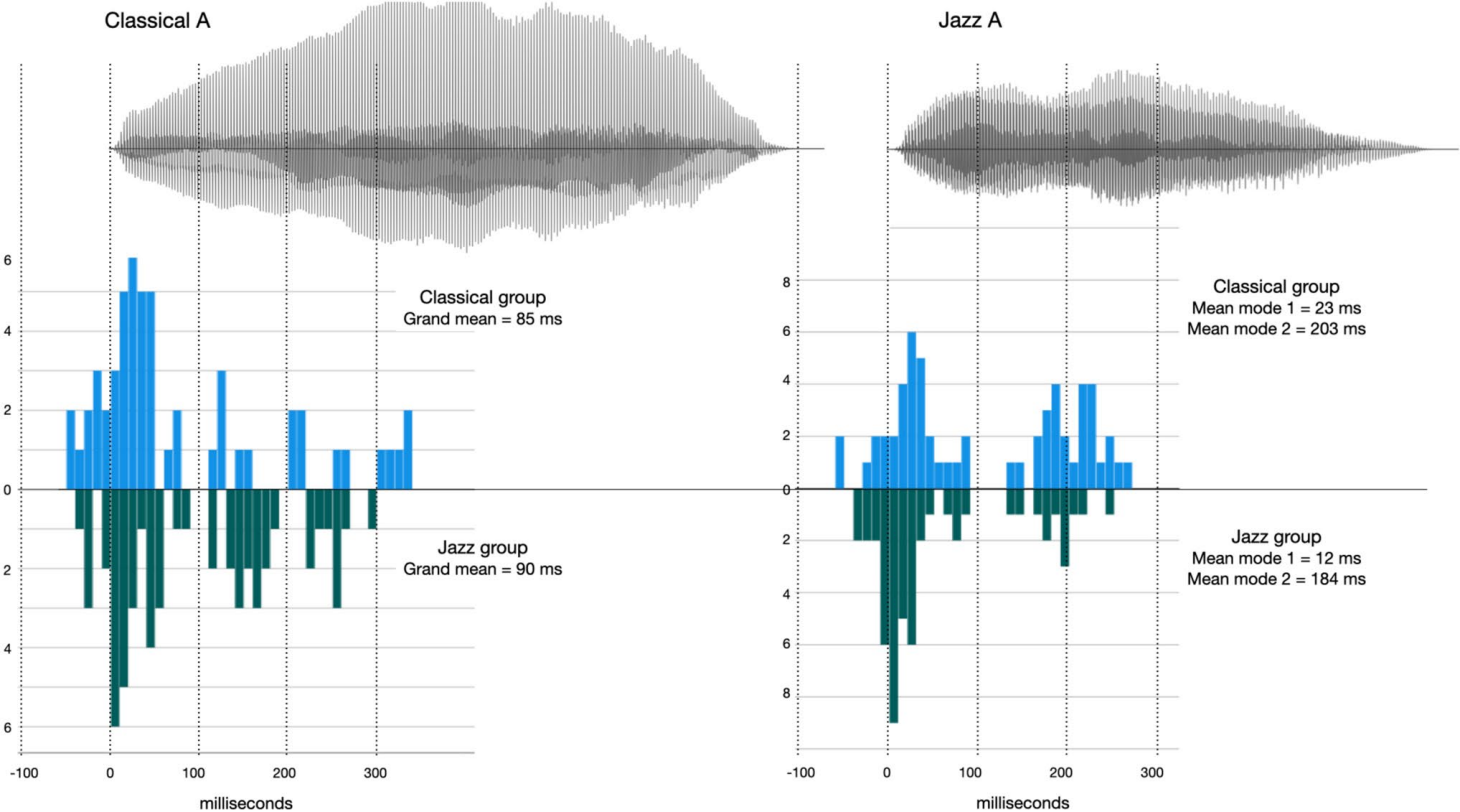
Waveform (amplitude/time) and histograms of responses to the Classical A and Jazz A stimulus sounds in the CLICK task in Experiment 1 for each of the two participant groups. Y-axis of histograms = frequency

In this section, we examine the responses to each stimulus sound in the click alignment task, with a particular focus on potential multimodal distributions. Appendix A8 (Online Supplemental Material) provides histograms of the responses of each group with normal distribution curves for all vocal stimuli. After manual inspections of the histograms, we decided to have a closer look at the apparent bimodal distribution of the Jazz A stimulus compared to the corresponding Classical A stimulus. Waveform representations of these sounds overlaid with histograms showing the distribution of all click trials for each of the two participant groups are given in Fig. 6. For the Classical A stimulus (i.e., “A” vocalized by a classical singer), the responses of both participant groups exhibit a unimodal distribution with an early peak and a long right tail (skewness = 1.086). While both participant groups have very similar numbers of participants with an early peak around 17 ms (34 Classical vs. 33 Jazz, within a time window ranging from − 42 to + 335 ms relative to sound onset, SD = 29 ms for both groups), the distribution of responses in the tail differs between the two groups, as the jazz musicians’ distribution is distinctly “clumpy” while the classical musicians responses are more uniform (see Fig. 6, left panel).

The histogram of the Jazz A stimulus (i.e., “A” vocalized by a jazz singer), on the other hand, shows clear bimodal distributions for both groups, with a split point at 100 ms. The locations of each modal peak (M1 around 20 and M2 around 200 ms, respectively) correspond to inflection points in the amplitude envelope of the sound (see Fig. 6, right panel). Forty-two trials by jazz participants belong to the first mode and 12 to the second. For the classical participants the number of trials at each mode is 28 and 27, respectively. There is also a difference in mean location between the two groups at each mode: At the first mode jazz is earlier by 11 ms, at the second mode jazz is earlier by 19 ms. Jazz participants’ P-centers are earlier than those of classical participants in two ways in response to this sound. First, most of the jazz participants (78%) place their P-centers around the first mode, while the classical participants are split almost perfectly evenly between the two modes. Second, relative to each mode, the P-centers of jazz participant sub-groups are always earlier than those of the classical participants. Thus, the overarching differences between our two participant groups persist even when presented with stimuli that afford more than one possible p-center location, though it is more complex than would be the case if jazz participants’ P-centers all (or nearly all) were simply clustered around the first mode, and the classical partcipants’ P-centers were around the second (see additional comments in *Discussion* below).

### Discussion

In Experiment 1, two groups of expert singers who perform jazz and classical music, respectively, were asked to synchronize either a click track or their tapping with claves to a set of looped vocal and non-vocal control stimuli. In addition to assessing the effect of their specific musical expertise on their perception of those stimuli, we sought to examine the effects of basic acoustic factors (initial bilabial consonant/direct vowel onset for vocal stimuli, and soft vs. sharp attack for non-vocal stimuli) and genre-based factors (whether vocal stimuli were sung by jazz or classical vocalists). The dependent measures were the mean location of the click or tap (P-center location), and the variability about that mean (P-center variability, which is also the width of the “beat bin” for sounds in a metrical context).

For the control stimuli, we found main effects of Attack (soft vs. sharp) on P-center location and variability for all participants in the click alignment task, and an effect on P-center location in the tapping task – sounds with a soft attack are later and more variable – replicating previous studies (Danielsen et al., 2019, 2022; Gordon, 1987; London et al., 2019; Villing, 2010; Vos & Rasch, 1981). As expected, there was no effect of Attack on tap variability. Our previous experiments using the same paradigm (Danielsen et al., 2019, 2022; London et al., 2019) have shown that the tap task is insensitive to P-center variability. The participants do only one series of tapping trials for each stimulus, and the motor activity in the task likely stabilizes the performance so that the variability caused by the differing beat bins of the stimuli is only manifest as inter-subjective differences in accuracy. Similar results have been obtained in other tapping studies as well (Will et al., 2015; Witek et al., 2020). Moreover, as was also expected, there were on average no significant differences between the two groups for P-center location with the control stimuli. However, there was significant interaction with Group so that the more complex fiddle sound produced a greater difference between the two participant groups, similar to the results reported by Danielsen and colleagues (2022). Moreover, the classical participants did have higher variability than jazz participants, but this occurred only in the tapping task.

In line with our hypotheses, we found a significant effect of participant background/training (classical vs. jazz) across all vocal stimuli for P-center location in the click alignment task (mean P-center locations 17 ms later for the classical participants compared to jazz), though not in the tapping task. As to variability, participant background/training showed a significant effect in the TAP task (4 ms higher for the classical compared to the jazz participant group). Albeit non-significant, the mean variability was also higher (9 ms, *p =*.087) for the classical participants in the click task. Thus, for the vocal stimuli, our primary hypotheses that jazz participants overall would have earlier P-center locations and lower variability in comparison to classical participants were both supported, though this finding is tempered by the more complex responses to the Jazz A stimulus (see discussion in the following paragraph). We also found the expected significant effects of stimulus vocal genre (i.e., whether the sounds were sung by a classical vs. a jazz vocalist). The P-centers for the classical vocal stimuli had on average a 72-ms (CLICK) and 86-ms (TAP) later P-center than the jazz stimuli, respectively. The variability for classical vocal stimuli was also higher overall – 4 ms (CLICK) and 3 ms (TAP), respectively.

We also examined the specific effects of different vocal stimuli. Our most interesting finding related to between-group differences comes from the responses to the “A” sounds. The Jazz A stimulus reaches its initial dynamic peak much faster than the Classical A, but there is a clear secondary peak in the Jazz A sound. This is reflected in the bimodal responses to the Jazz A by both groups of participants. The two modes apparent in the histograms for the Jazz A sound (see Fig. 6) correspond to inflection points within the amplitude envelope for the sound. Thus, when asked to “find the beat” in this stimulus, our participants made use of the different affordances in this complex sound as temporal targets for their responses. As expected, given the aesthetic preferences of the two genres, most jazz participants tended to place their P-centers around the first mode, and they have also earlier P-centers at each mode when compared to the classical participants.

From this experiment, a question naturally arose regarding the perception of these sounds in a spoken as opposed to a sung/musical context, which brings us to Experiment 2.

## Experiment 2

### Methods

#### Participants

Professional female singers (14 jazz, 13 classical) were recruited from the Oslo area (age range 21–60 years; average age 27.6 years). Six of the classical and seven of the jazz singers also participated in Experiment 1. Written informed consent was obtained and each participant received a gift card (approx. value 20 USD) for their participation in the experiment.

#### Stimuli

The sung stimuli consisted of looped/repeated presentations of the syllables “A” and “E” from Experiment 1. Additionally, they included spoken versions of “A” and “E” produced by the same singers that created the stimuli used in Experiment 1, plus syllables “A” and “E” spoken by a female without any experience or training as a singer. A click-on-click alignment task was included as a basic check of the participants’ ability to perform the experimental task; data from the click trials were not analyzed. The final set of sounds analyzed in the experiment are given in Table 3 below.

**Table 3:** Sounds used in Experiment 2

Stimulus genre	Sung		Spoken	
Classical vocal stimuli	“A”	“E”	“A”	“E”
Jazz vocal stimuli	“A”	“E”	“A”	“E”
Neutral vocal stimuli			“A”	“E”

#### Procedure, apparatus, and setup

In Experiment 2, the participants did click alignment trials only. The procedure, apparatus, and setup were the same as in Experiment 1. In total 750 click locations (10 stimuli × 3 trials × 25 participants) were recorded. P-centers are reported in milliseconds relative to the physical onset of each stimulus. Eight trials were excluded because the click occurred more than 80 ms before the onset of the sound. The standard deviations of the three trials for each stimulus for each participant were also calculated. The grand averages of participants’ P-centers and standard deviations were used as measures of the overall CLICK P-center location and variability for each stimulus. After the experimental task, participants completed a background questionnaire. Participants were encouraged to proceed through the experiment at their own pace and to take breaks as needed. One of the experimenters waited nearby should any questions/problems arise.

#### Statistical analysis

We used a Linear Mixed Model with Group as between-subjects and Stimulus Genre (classical, jazz, neutral) and Performance Mode (sung or spoken) as within-subjects fixed effects (see Table 3). Because we expected differences in the effect of Stimulus Genre between groups, a Group–Stimulus Genre interaction was added to the model. In line with our hypotheses, we also added interaction between Stimulus Genre and Performance Mode. Participant was included as Random Intercept. P-center (e.g., click location relative to stimulus onset) and variability (average click location for each stimulus per participant) were the dependent variables. Model fit was assessed by comparing AIC scores for full models to a null model with Intercept and Random Intercept (Participant), only. The analyses were performed using Satterthwaite approximations (Satterthwaite, 1946), restricted maximum likelihood estimation (REML), and a variance components covariance matrix. Residuals were normally distributed. Follow-up paired-samples t-tests of differences between the five stimulus categories (sung classical, sung jazz, spoken classical, spoken jazz, spoken neutral) were performed with Bonferroni correction for multiple comparisons. All analyses were performed in SPSS 29.

### Results

**Fig. 7:**
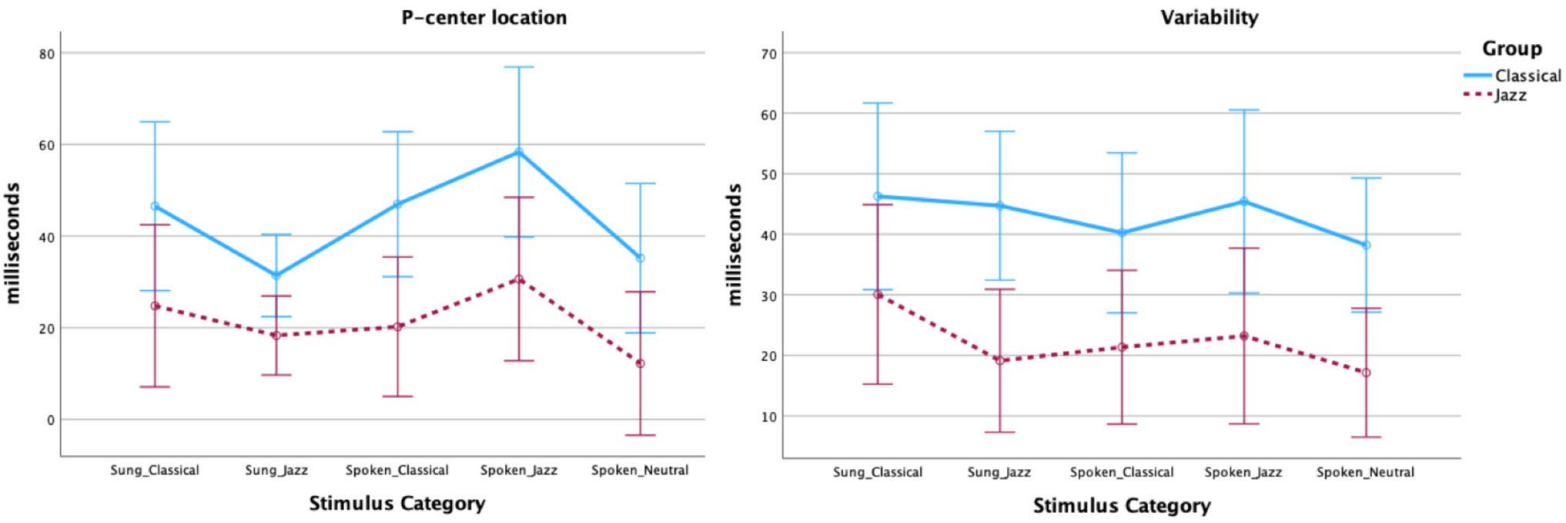
Plots of mean P-center location relative to stimulus onset (**top**) and mean variability (**bottom**) for each sound in the Sung Classical, Sung Jazz, Spoken Classical, Spoken Jazz, and Neutral Stimulus Genres in the click alignment task in Experiment 2, separated by participant genres. Error bars are 95% confidence intervals

Descriptive statistics of the mean location and variability (per stimulus) for click alignment and tapping trials for all stimulus categories are given in Table 4. (A complete account of the results of the linear mixed models and paired-samples t-tests is provided in Tables A6 and A7 in the Appendix (Online Supplemental Material)). Plots of results for each sound in the click alignment task are shown in Fig. 7.

**Table 4:** Summary statistics for P-center location and variability in milliseconds for all stimulus sounds and categories in the click alignment task in Experiment 2. P-center means and variability are reported as mean of mean locations and mean of standard deviations for each participant’s three trials of each stimulus

	Sung						Spoken					
Stimulus/Group	P-center mean			Variability			P-center mean			Variability		
	Class.	Jazz	Total	Class.	Jazz	Total	Class.	Jazz	Total	Class.	Jazz	Total
Classical A	55	35	45	44	32	38	44	16	29	49	23	35
Classical E	38	14	26	48	28	38	50	24	37	32	20	26
Avg. Classical	47	25	35	46	30	38	47	20	33	40	21	30
Jazz A	24	17	20	47	24	35	52	20	35	47	23	35
Jazz E	39	20	29	43	15	28	65	42	53	44	23	33
Avg. Jazz	32	18	25	45	19	31	58	31	44	46	23	34
Neutral A							36	10	22	35	23	28
Neutral E							34	15	24	42	12	26
Avg. Neutral							34	12	23	38	17	27

We found a significant difference in P-center location (*p =*.044) between the two participant groups, as the classical participants had on average 22 ms later P-center locations (95% CI: 0–44) than the jazz participants. There was also a significant effect of the within-subjects factor Stimulus Genre but no significant interaction. The P-centers of stimuli produced by a classical singer were overall 30 ms later (*p <*.001) and jazz singers 22 ms later (*p <*.001) than stimuli produced by a neutral speaker. Whether the stimuli were sung or spoken also had a significant effect (*p <*.001). There was also a significant interaction between Stimulus Genre and Performance Mode, see plots in Fig. 7 and Table A6 in the Appendix (Online Supplemental Material). Post hoc pairwise comparisons (Bonferroni corrected) show significant differences between the following stimulus categories: sung and spoken jazz (*p =*.023) and spoken jazz and spoken neutral (*p =*.018).

The linear mixed model for variability showed a significant difference (*p =*.025) between the two participant groups, as the classical participants had on average 21 ms higher variability (95% CI: 3–40) than the jazz participants. There was a significant effect of the within-subjects factor Stimulus Genre at the level Classical stimuli (*p =*.047). Classical stimuli had overall 15 ms higher and Jazz 5 ms higher variability than Neutral stimuli. There was no significant interaction between Stimulus Genre and Group. Whether the stimuli were sung or spoken neither had a significant effect and nor any interaction between Performance Mode and Stimulus Genre. None of the post hoc pairwise comparisons of stimulus categories were significant.

### Discussion

In Experiment 2, we wanted to investigate whether the group differences found in Experiment 1 persist beyond singing to syllables spoken by the singers trained in the two genres and a non-singer. As expected, we replicated the between-group differences for P-center location and variability. Surprisingly, however, there were no interaction effects involving group, which means that the between-group differences persisted when syllables were spoken by singers or by a speaker without vocal training. This unexpected result could be explained by the click alignment task being inherently musical: Even when the target sound is a spoken syllable the looped stimuli might resemble music in a way that activates the participants’ musical expertise.

As expected, the syllables sung by the classical singer produce later P-centers and higher variability in both participant groups. Interestingly, however, the picture is reversed for the spoken syllables, where syllables spoken by the jazz singer have later P-centers and higher variability than the syllables spoken by the classical singers (for the classical syllables sung and spoken P-center is approximately the same, whereas for jazz the difference between sung and spoken is significant, *p =*.023). One possible explanation could be that the click in the recording situation had a different effect on the two singers. The spoken neutral syllables had an earlier P-center than the other conditions, which might be explained by the neutral syllables being more typical of speech because of this speaker’s lack of experience with singing. The sung classical syllables produced higher variability than the sung jazz syllables for the jazz participants. This is in line with Experiment 1 and what one would expect given jazz singers’ frequent interaction with percussive comp instruments, which requires more narrow beat bins.

### General discussion and conclusion

We investigated the perception of P-centers in sung and spoken syllables by two groups of participants, expert vocalists in jazz and classical music, respectively. The stimuli consisted of a variety of control, sung, and speech sounds, repeated at an isochronous 750-ms interval, which falls within the range for optimal beat entrainment (80 BPM). Participants had to perform two synchronization tasks, either aligning a click-track or tapping along with clave sticks to each stimulus. In the first experiment participants were presented with control stimuli and with sung stimuli (performed by expert jazz and classical vocalists). In the second experiment, participants were presented with a subset of the sung stimuli from Experiment 1, along with matched spoken versions of the same stimuli, as well as spoken versions performed by a female speaker without vocal training.

Our results from the first experiment broadly support our experimental hypotheses, though not without some interesting complications. As per our first set of hypotheses, participant responses to the control stimuli replicated our previous work; we found that stimuli with soft attack/slower rise times had later P-centers and greater variability than control stimuli with faster rise times, and there was no difference between participant groups in terms of P-center location. There was no difference between groups in terms of P-center variability in the click alignment task, though there was a small difference in variability in the tapping task, as classically-trained participants had slightly higher variability than jazz-trained participants. As per our hypotheses for the vocal stimuli, jazz participants had significantly earlier P-centers than classical participants in the click alignment task, though not in the tapping task. Moreover, and in line with the control stimuli, classically-trained participants had slightly higher tapping variability (4 ms) than jazz-trained participants. Finally, for all participants the vocal stimuli produced by a classical singer had later P-centers and higher variability than the stimuli produced by a jazz singer, as per our final set of hypotheses for Experiment 1. For the second experiment our hypotheses were that (a) we would replicate the results of Experiment 1 for the sung stimuli, and (b) the between-group differences for sung stimuli would not be evident for the spoken stimuli. The first hypothesis was supported, but the second was not.

### Between-group differences with sung and spoken stimuli: Top-down influences on P-center perception, and the effect of task demands

In both experiments there were clear between-group differences in the perception of vocally produced sounds, confirming our core experimental hypotheses. What might be the origin and neural bases for these differences in P-center location and variability? We believe the differences originate in subtle but significant differences in the motor patterns for vocal articulation of our two participant groups, and that those top-down models modulate bottom-up sensory inputs, as well as influence how those inputs are processed. Rimmele and colleagues (2018) provide evidence that top-down phase resetting driven by the motor system facilitates bottom-up perceptual processing, and Morillon et al. (2014) note that top-down rhythmic motor routines can sharpen sensory representations, though we would say “shape” rather than “sharpen,” as the optimization of the sensory representation for a P-center may not necessarily involve a narrower beat bin (see also Molinari et al., 2007). Cannon and Patel (2021), building upon the Action Simulation for Auditory Prediction (ASAP) hypothesis (Patel & Iversen, 2014), have detailed the neural pathways in beat perception, and argue that the supplementary motor area may be driving auditory predictions (in cerebellar regions) via the dorsal auditory pathway using more flexible models in the premotor and parietal cortex. In the case of expert musicians, and with the right contextual cues, models based on very specific sensorimotor representations of particular music-producing actions could be activated.

In becoming experts, musicians develop highly specific motor representations of their own actions and use those models when singing and playing – especially when playing with others, as anticipatory and error-correction mechanisms must be involved in order to maintain group synchrony. For instrumentalists, those motor representations are of the bodily actions involved in playing their musical instruments (Van Der Steen & Keller, 2013). For singers, these forward models involve the use of their vocal phonating and articulatory mechanisms – and hence there is a great overlap with speech production (Kotz, 2022; Rauschecker & Scott, 2016; Tian & Poeppel, 2010). The differences observed here between classical and jazz singers, as well as with instrumentalists in our previous studies, are probably due to consistent differences in their motor representations/forward models used when performing. These fine-grained differences emerge due to the extremely high levels of training that these models have received. Crucially, this training involves motor actions in response to both particular kinds of sounds and a particular usage of those sounds, based upon the musical/aesthetic preferences of their particular genre and style. Pearce (2015) details how expertise improves the predictive abilities of musicians, both in terms of their ability to process larger chunks of information and having a greater awareness of details. Concomitant with this awareness of detail is a sharpening of experts’ low-level (fine-grained) perception of the elements within those larger chunks of information, especially in relation to their means of production.

The influence of top-down motor representations in the processing of relevant temporal information may also explain why the between-group differences persisted in Experiment 2 in the spoken syllable context, but not with the control sounds in Experiment 1, and also why the between-group differences were more apparent in the click alignment task than in the tapping task. In both of our experiments the context is an essentially musical one: synchronizing a sound under the control of the participant with a repeated target sound (i.e., a very simple rhythm that gives rise to a sense of beat or pulse). Our different experimental tasks, however, engaged different motor effectors, and as a result, different aspects of our participants’ musical training and enculturation.

The control sounds in Experiment 1 were not related to any relevant means of sound production for our participants, as they were either wholly artificial (clicks and noise bursts) or a musical sound unrelated to their own way of making music (a bowed fiddle). Thus, while the task remained musical (synchronization with a sound), the targets were not relevant to vocal production. All the other stimuli in both experiments, however, were vocal sounds: singing or speaking. In these trials, our participants used their relevant motor representations to guide their responses, in so far as they were able. In the click alignment trials their vocally-based sensorimotor representations were available as they performed the experimental task, and hence the between-group differences emerged. These differences persisted even with spoken stimuli, as the context (synchronizing with a rhythmically repeated syllable) invites – indeed, demands – the use of their relevant motor representations, representations grounded in their musical training. In the tapping task, however, a different motor representation was required – using the arms as effectors to play clave sticks in synchrony with the stimuli. In this case, a more generic motor representation was probably in play, something of the sort that Cannon and Patel (2021) describe in their model.

The match or mismatch between stimulus (artificial sound, instrumental sound, vocal sound), task (tapping vs. click alignment) and participant expertise may be why previous research has found little differences between musicians in a generic rhythm task. Tellingly, in the research cited above, the one difference between musician groups that does emerge is that between percussionists versus other instrumentalists, and of course tapping tasks (with or without the use of a drumstick or similar) are directly related to a drummer's motor representations. For other musicians, generic tapping tasks do not afford use of their particular motor representations for (musical) rhythm production, and the fine-grained differences that might emerge as a result are thus not captured (Manning & Schutz, 2016; Matthews et al., 2016; Nikjeh et al., 2009).

### Speech sounds are complex; sung speech even more so

Finding the P-centers in speech sounds is not a straightforward affair. While it is generally agreed that P-centers are based upon the recovery of articulatory information (Fowler, 1979, 1983) – that is, grasping the motor sequence that produces the sound – no single articulatory landmark serves as the basis for P-center location (de Jong, 1994; Patel et al., 1999). Franich (2018), in a study of Bantu (a complex tone language whose tones function differently from Asian languages) found an interaction between tone and P-center location. Thus, we should not be surprised that in singing the interactions between linguistic and musical features create an even more complex matrix for the determination of P-center location and other characteristics. In addition to the structure of the initial consonant (determined by tongue and jaw position), voicing, and vowel position, singing adds more subtle onset characteristics, a dynamic envelope of the attack, stabilization of the steady-state pitch/tone and vibrato, and additional timbral effects (Lindblom & Sundberg, 2014).

In other words, sung syllables are capable of providing multiple affordances for P-center locations, and thus it should not surprise us that both (a) for some sung sounds, performers from different styles will gravitate to particular locations/affordances based upon their stylistic practice and aesthetic goals (e.g., “tight” placement on the beat near to an initial articulatory landmark, or a “looser”/more relaxed placement relative to the stabilization of vibrato), and (b) for other sounds, there may even be multiple affordances for performers within a given style, providing options for their personal aesthetic preferences and/or contextual influences (e.g., the jazz A sound).

### Enculturation and affordances: Listening to versus listening through

As noted above, when any sound is presented in a rhythmic/repeated fashioned, and a musician is asked to synchronize with it, that context/cue engages their musical expertise to the extent that it is relevant and helpful. Any interpersonal synchronization task, including and especially a group musical performance, is a form of joint action (Keller et al., 2014; Sebanz & Knoblich, 2009). As such, the musical task involves the coordination of both the sounds each musician produces, and also a coordination of the actions involved in producing those sounds. As a result, musicians both listen *to* the sounds they make (and the sounds of others), as well as *through* the sounds they hear to the actions which produce them. The “to” versus “through” forms of listening have been discussed both in musical aesthetics (Ihde, 2012; Schaeffer, 2017; Scruton, 1999) as well as in ecological attention and perception (Clarke, 2005; Gaver, 1993a, b). When listening in a “through” mode, we not only attend to the sound source, but specifically to what it is doing/how it is being used to create the sounds we hear – hence we are aware of striking, blowing, scraping, and all the other actions that give rise to sound. Our data suggest that musicians, even when given a seemingly acousmatic problem in the sense that the sounds are putatively divorced from their source(s) of production (Schaeffer, 2017), nonetheless attend to aspects of its production in the context of a synchronization task. That is, musicians, and especially expert musicians, are always listening both “through” and “to” sounds when those sounds are produced by actions that they themselves make as trained performers. Indeed, it is in the context of listening to and playing with other musicians that musical experts develop their expertise. In our two experiments the vocal stimuli presented not only acoustic cues for synchronization/P-center location, but also cues regarding their means of vocal production. Thus, even though our participants were not speaking or singing in synchrony with the target sounds, and certainly not performing with other musicians, the vestiges of joint action that remained in our experimental context were enough to engage their top-down sensorimotor models, as would be used in an actual music-performance context.

### Directions for future research

Our results in this experiment, and in our previous investigations of the effect of expertise on P-center perception, suggest several lines for future research. Thus far, we have restricted the presentations of our stimuli to a narrow range of tempos anchored around 1.5 Hz (600–800 ms interstimulus interval (ISI), or 80–100 BPM), near the range in which beat perception is strongest (for a summary of research, see Levitin et al., 2018; London, 2012). However, as there are neural dynamics involved with the top-down modulation of sensory and sensorimotor signals and activity (Rimmele et al., 2018), there may be a rate limit for such modulations. Therefore, a series of experiments with progressively faster ISIs, with the stimuli modified accordingly, may show a limit to the effect of musical expertise. That is, at faster rates, between-group differences would disappear, as the cues for synchronization/P-center location would be wholly stimulus driven. We speculate this might well occur around the 250- to 300-ms ISI range, where the sense of beat becomes attenuated, even though we are still able to synchronize our actions with stimuli at those faster rates (Repp, 2005; Repp & Su, 2013).

To the extent that top-down sensorimotor models are based upon expert musicians’ highly developed motor programming related to their particular instruments, there may be instrument-specific differences which would interact with the genre/stylistic differences between the participant groups studied here. As we did not control for musical instrument in our first experiment, an experimental design which specifically crosses instrument (e.g., piano vs. violin, or guitar vs. saxophone) and genre (jazz vs. classical) in a 2 × 2 factorial design could show which factor may be dominant. This experiment might also involve EEG and/or a TMS component to find more specific evidence regarding the involvement of the motor system in these tasks. Finally, to close our investigation of expertise with respect to vocalists, we have been exploring ways to probe P-center perception of speech sounds with an experimental task that does not involve the rhythmic/isochronous presentation of stimuli (i.e., a context that is not even quasi-musical). With such a method, we would hope to create a context in which our second hypothesis for our second experiment holds true: where musical training is not relevant, other forms of training/expertise (e.g., basic linguistic knowledge) and information (i.e., acoustic cues) become dominant, and between-group differences in P-center perception would disappear.

Our broader takeaway is that rhythmic expertise is not a simple manifestation of “being more precise,” as is typically measured in terms of lower variability and/or tighter synchrony between a temporal target and participant response. Here and in our previous study, we found the opposite, in some sense. Musical expertise is ultimately manifested in the musician’s ability to make aesthetically appropriate choices in specific musical contexts and the expressive and social goals of their performances. These manifestations of expertise are not readily studied in the laboratory, but will require data gathered in richer, more ecologically valid contexts, along with more sophisticated ways of analyzing the data (ways that may involve a combination of both rhythmic and non-rhythmic factors, such as dynamics, timbral manipulation, intonation, and so forth). Vocal music has been under-studied in the music perception literature, and understandably so, given the difficulties and complexities involved. But as most of the worlds’ music involves songs and singing (Mehr et al., 2019), it is an area of music perception ripe for further study.

## Supplementary information

Below is the link to the electronic supplementary material.

Supplementary file1 (PDF 494 KB)

## Data Availability

The datasets generated during the current study will be made available upon publication. None of the experiments were preregistered.

## Footnotes

1 In both experiments, the jazz participants included singers with extensive experience in related styles, such as blues, soul, and R&B.

2 Here and below in the *Results* section we refer to the vocal stimuli with an initial “M” sound as having soft attacks, and vocal stimuli with a direct vowel onset as having sharp attacks; as is noted in the *Discussion* section, the attack phase of these sounds is complex, and thus the distinction is not wholly accurate. For convenience, however, we use the sharp/soft designations to note broad similarities between the vocal versus neutral stimuli.
