# Supplementary material for: The influence of vocal expertise on the perception of microrhythm in song and speech
Source: Atten Percept Psychophys. 2025 Apr 25;87(5):1750–70. doi: 10.3758/s13414-025-03057-y (PMC12204903; doi:10.3758/s13414-025-03057-y)
Supplement: Supplementary file 1 — Supplementary file1 (PDF 494 KB) [file 13414_2025_3057_MOESM1_ESM.pdf]

Online Supplemental Material

Appendices

Table A1. Estimates of fixed effects of Group and Attack and Random Intercept (Participant) on P-center location and variability in response to the Control stimuli in Experiment 1.

CLICK LOCATION (Full model AIC = 4601; Null model: Intercept + Random Intercept (Participant) AIC = 4729)

Fixed Effects						95% Confidence Interval	
Parameter	Estimate	Std. Error	df	t	Sig.	Lower Bound	Upper Bound
Intercept	1.047	6.044	52.256	.173	.863	-11.081	13.174
Group=Classical	9.491	7.690	34.386	1.234	.226	-6.131	25.113
Group=Jazz	0	0	.	.	.	.	.
Control stimuli - soft attack	61.588	5.348	386.846	11.515	<.001	51.072	72.103
Control stimuli - sharp attack	0	0	.	.	.	.	.
Random Effects						95% Confidence Interval	
Parameter	Estimate	Std. Error	Wald Z		Sig.	Lower Bound	Upper Bound
Intercept (Participant) Variance	274.511	129.527	2.119		.034	108.874	692.141

CLICK VARIABILITY (Full model AIC = 1376; Null model: Intercept + Random Intercept (Participant) AIC = 1406)

Fixed Effects						95% Confidence Interval	
Parameter	Estimate	Std. Error	df	t	Sig.	Lower Bound	Upper Bound
Intercept	12.736	4.870	56.384	2.615	.011	2.981	22.490
Group=Classical	7.296	6.025	34.011	1.211	.234	-4.948	19.541
Group=Jazz	0	0	.	.	.	.	.
Control stimuli - soft attack	21.136	4.719	107.000	4.479	<.001	11.782	30.491
Control stimuli - sharp attack	0	0	.	.	.	.	.
Random Effects						95% Confidence Interval	
Parameter	Estimate	Std. Error	Wald Z		Sig.	Lower Bound	Upper Bound
Intercept (Participant) Variance	126.339	83.839	1.507		.132	34.410	463.869

TAP LOCATION (Full model AIC = 1401; Null model: Intercept + Random Intercept (Participant) AIC = 1465)

Fixed Effects						95% Confidence Interval	
Parameter	Estimate	Std. Error	df	t	Sig.	Lower Bound	Upper Bound
Intercept						-12.011	11.836
Group=Classical	4.632	6.966	33.139	.665	.511	-9.539	18.803
Group=Jazz	0	0	.	.	.	.	.
Control stimuli - soft attack	52.795	6.577	103.569	8.027	<.001	39.752	65.838
Control stimuli - sharp attack	0	0	.	.	.	.	.
Random Effects						95% Confidence Interval	
Parameter	Estimate	Std. Error	Wald Z		Sig.	Lower Bound	Upper Bound
Intercept (Participant) Variance	354.197	124.217	2.851		.004	178.126	704.306

Table A2. Main effects of the Mixed RM ANOVA for P-center variability in the tapping task (TAP) in response to the Control stimuli in Experiment 1. Participant Groups: Classical vs. Jazz. Attack: Sharp versus Soft. Stimulus: Short vs. Long.

TAP VARIABILITY				
Tests of Between-Subjects Effects	df	F	Sig.	η_p^2
Group	1	6.219	.018	0.159
Error (Group)	33			
Tests of Within-Subjects Effects	df	F	Sig.	η_p^2
Attack	1	.466	.499	.014
Attack * Group	1	.019	.891	.001
Error(Attack)	33			
Stimulus	1	.184	.670	.006
Stimulus * Group	1	.237	.629	.007
Error(Stimulus)	33			
Attack * Stimulus	1	33.665	<.001	.505
Attack * Stimulus * Group	1	5.418	.026	.141
Error(Attack * Stimulus)	33			

Table A3. Results of models with fixed effects of Group and Attack(Stimulus Genre) and Participant as Random Intercept for Vocal stimuli in Experiment 1.

CLICK LOCATION (Model fit: Intercept + Random Intercept (Participant) AIC = 9993; Full model AIC = 9439)

Fixed Effects						95% Confidence Interval	
Parameter	Estimate	Std. Error	df	t	Sig.	Lower Bound	Upper Bound
Intercept	44.595	6.505	72.764	6.856	<.001	31.630	57.559
Group=Classical	17.094	7.570	33.675	2.258	.031	1.704	32.484
Group=Jazz	0	0	.	.	.	.	.
Classical stimuli - soft attack	135.610	6.061	809.827	22.375	<.001	123.713	147.506
Classical stimuli - sharp attack	-3.012	6.026	809.883	-.500	.617	-14.839	8.816
Jazz stimuli - soft attack	7.219	6.069	810.111	1.189	.235	-4.694	19.132
Jazz stimuli - sharp attack	0	0	.	.	.	.	.

Random Effects						95% Confidence Interval	
Parameter	Estimate	Std. Error	Wald Z		Sig.	Lower Bound	Upper Bound
Intercept (Participant) Variance	370.286	127.147	2.912		.004	188.910	725.803

CLICK VARIABILITY (Model fit: Intercept + Random Intercept (Participant) AIC = 2819; Full model AIC = 2731)

Fixed Effects						95% Confidence Interval	
Parameter	Estimate	Std. Error	df	t	Sig.	Lower Bound	Upper Bound
Intercept	35.770	4.677	99.780	7.648	<.001	26.491	45.049
Group=Classical	8.599	4.950	33.903	1.737	.091	-1.461	18.659
Group=Jazz	0	0	.	.	.	.	.
Classical stimuli - soft attack	-11.111	5.069	247.862	-2.192	.029	-21.095	-1.127
Classical stimuli - sharp attack	-7.944	5.069	247.862	-1.567	.118	-17.928	2.039
Jazz stimuli - soft attack	11.559	5.088	248.110	2.272	.024	1.538	21.581
Jazz stimuli - sharp attack	0	0	.	.	.	.	.

Random Effects						95% Confidence Interval	
Parameter	Estimate	Std. Error	Wald Z		Sig.	Lower Bound	Upper Bound
Intercept (Participant) Variance	109.476	53.994	2.028		.043	41.639	287.828

TAP LOCATION (Model fit: Intercept* AIC = 3329; Full model AIC = 2884)

Fixed Effects						95% Confidence Interval	
Parameter	Estimate	Std. Error	df	t	Sig.	Lower Bound	Upper Bound
Intercept	25.227	6.417	70.129	3.931	<.001	12.429	38.026
Group=Classical	6.179	7.521	34.010	.822	.417	-9.104	21.463
Group=Jazz	0	0	.	.	.	.	.
Classical stimuli - soft attack	163.214	5.866	249.000	27.825	<.001	151.661	174.766
Classical stimuli - sharp attack	5.070	5.866	249.000	.864	.388	-6.482	16.623
Jazz stimuli - soft attack	14.245	5.866	249.000	2.429	.016	2.692	25.798
Jazz stimuli - sharp attack	0	0	.	.	.	.	.
Random Effects						95% Confidence Interval	
Parameter	Estimate	Std. Error	Wald Z		Sig.	Lower Bound	Upper Bound
Intercept (Participant) Variance	354.197	124.217	2.851		.004	178.126	704.306

*The null model with Intercept + Participant did not converge.

TAP VARIABILITY (Model fit: Intercept + Random Intercept (Participant) AIC = 2041; Full model: AIC = 2015)

Fixed Effects						95% Confidence Interval	
Parameter	Estimate	Std. Error	df	t	Sig.	Lower Bound	Upper Bound
Intercept	15.525	1.397	68.545	11.111	<.001	12.738	18.313
Group=Classical	4.445	1.648	34.008	2.698	.011	1.097	7.794
Group=Jazz	0	0	.	.	.	.	.
Classical stimuli - soft attack	2.711	1.259	249.000	2.153	.032	.231	5.192
Classical stimuli - sharp attack	.935	1.259	249.000	.743	.458	-1.545	3.416
Jazz stimuli - soft attack	3.684	1.259	249.000	2.925	.004	1.204	6.165
Jazz stimuli - sharp attack	0	0	.	.	.	.	.
Random Effects						95% Confidence Interval	
Parameter	Estimate	Std. Error	Wald Z		Sig.	Lower Bound	Upper Bound
Intercept (Participant) Variance	17.297	5.960	2.902		.004	8.804	33.983

Table A4. Results of models with fixed effects of Group and Sound Type(Stimulus Genre) and Random Intercept (Participant) for Vocal stimuli in Experiment 1.

CLICK LOCATION (Full model AIC = 9339; Null model: Intercept + Random Intercept (Participant) AIC = 9993)

Fixed Effects						95% Confidence Interval	
Parameter	Estimate	Std. Error	df	t	Sig.	Lower Bound	Upper Bound
Intercept	24.868	7.605	129.247	3.270	.001	9.823	39.914
Group=Classical	17.016	7.608	33.689	2.237	.032	1.549	32.483
Group=Jazz	0	0	.	.	.	.	.
Classical Ma	132.798	8.163	805.794	16.269	<.001	116.776	148.821
Classical Me	18.161	8.125	805.801	2.235	.026	2.213	34.108
Classical A	178.204	8.184	806.055	21.775	<.001	162.139	194.268
Classical E	15.346	8.125	805.801	1.889	.059	-.602	31.293
Jazz Ma	48.317	8.225	806.241	5.874	<.001	32.172	64.463
Jazz Me	39.920	8.184	806.111	4.878	<.001	23.856	55.985
Jazz A	6.449	8.143	805.690	.792	.429	-9.535	22.432
Jazz E	0	0	.	.	.	.	.

Random Effects						95% Confidence Interval	
Parameter	Estimate	Std. Error	Wald Z		Sig.	Lower Bound	Upper Bound
Intercept (Participant) Variance	370.286	127.147	2.912		.004	188.910	725.803

CLICK VARIABILITY (Full model AIC = 2731; Null model: Intercept + Random Intercept (Participant) AIC = 2819)

Fixed Effects						95% Confidence Interval	
Parameter	Estimate	Std. Error	df	t	Sig.	Lower Bound	Upper Bound
Intercept	26.240	5.769	179.454	4.548	<.001	14.856	37.623
Group=Classical	8.688	4.929	33.937	1.763	.087	-1.330	18.705
Group=Jazz	0	0	.	.	.	.	.
Classical Ma	-.750	6.952	243.904	-.108	.914	-14.444	12.944
Classical Me	.306	6.952	243.904	.044	.965	-13.388	13.999
Classical A	-2.500	6.952	243.904	-.360	.719	-16.194	11.194
Classical E	2.778	6.952	243.904	.400	.690	-10.916	16.472
Jazz Ma	33.528	7.005	244.380	4.786	<.001	19.730	47.326
Jazz Me	18.972	6.952	243.904	2.729	.007	5.278	32.666
Jazz A	8.917	6.952	243.904	1.283	.201	-4.777	22.611
Jazz E	0	0	.	.	.	.	.

Random Effects						95% Confidence Interval	
Parameter	Estimate	Std. Error	Wald Z		Sig.	Lower Bound	Upper Bound
Intercept (Participant) Variance	109.476	53.994	2.028		.043	41.639	287.828

TAP LOCATION (Full model AIC = 2805; Null model: Intercept* AIC = 3329)

Fixed Effects						95% Confidence Interval	
Parameter	Estimate	Std. Error	df	t	Sig.	Lower Bound	Upper Bound
Intercept	15.379	7.263	107.177	2.117	.037	.981	29.777
Group=Classical	6.179	7.521	34.005	.822	.417	-9.105	21.463
Group=Jazz	0	0	.	.	.	.	.
Classical Ma	148.057	7.479	245.001	19.796	<.001	133.326	162.789
Classical Me	19.569	7.479	245.001	2.617	.009	4.838	34.301
Classical A	198.066	7.479	245.001	26.483	<.001	183.335	212.797
Classical E	10.267	7.479	245.001	1.373	.171	-4.464	24.999
Jazz Ma	34.757	7.479	245.001	4.647	<.001	20.026	49.488
Jazz Me	19.696	7.479	245.001	2.634	.009	4.965	34.428
Jazz A	13.429	7.479	245.001	1.796	.074	-1.302	28.161
Jazz E	0	0	.	.	.	.	.

Random Effects					95% Confidence Interval	
Parameter	Estimate	Std. Error	Wald Z	Sig.	Lower Bound	Upper Bound
Intercept (Participant) Variance	383.207	123.980	3.091	.002	203.256	722.477

*The null model with Intercept + Random Intercept (Participant) did not converge.

TAP VARIABILITY (Full model AIC = 2003; Null model: Intercept + Random Intercept (Participant) AIC = 2041)

Fixed Effects						95% Confidence Interval	
Parameter	Estimate	Std. Error	df	t	Sig.	Lower Bound	Upper Bound
Intercept	15.540	1.663	122.841	9.344	<.001	12.247	18.832
Group=Classical	4.445	1.648	34.009	2.698	.011	1.097	7.794
Group=Jazz	0	0	.	.	.	.	.
Classical Ma	3.003	1.794	245.001	1.674	.095	-.531	6.538
Classical Me	1.311	1.794	245.001	.730	.466	-2.224	4.845
Classical A	2.391	1.794	245.001	1.333	.184	-1.143	5.925
Classical E	.532	1.794	245.001	.296	.767	-3.003	4.066
Jazz Ma	3.885	1.794	245.001	2.165	.031	.351	7.419
Jazz Me	-.028	1.794	245.001	-.016	.988	-3.562	3.506
Jazz A	3.456	1.794	245.001	1.926	.055	-.078	6.990
Jazz E	0	0	.	.	.	.	.

Random Effects					95% Confidence Interval	
Parameter	Estimate	Std. Error	Wald Z	Sig.	Lower Bound	Upper Bound
Intercept (Participant) Variance	17.191	5.961	2.884	.004	8.712	33.922

Table A5. Pairwise comparisons of Groups per Stimulus Category in Experiment 1. Significant results in **bold**. Bonferroni corrected for multiple comparisons.

CLICK LOCATION

Stimulus Category	Mean Difference Classical – Jazz Participants	Std. Error	Sig.	95% Confidence Interval Lower Bound	95% Confidence Interval Upper Bound
Classical Stimuli	8.083	8.600	.354	-9.394	25.561
Jazz Stimuli	26.750	9.240	**.007**	7.972	45.528
Vocal Stimuli	17.417	7.323	**.023**	2.535	32.298
Control Stimuli	10.819	7.460	.156	-4.342	25.981

CLICK VARIABILITY

Stimulus Category	Mean Difference Classical – Jazz Participants	Std. Error	Sig.	95% Confidence Interval Lower Bound	95% Confidence Interval Upper Bound
Classical Stimuli	7.768	5.939	.200	-4.301	19.837
Jazz Stimuli	9.835	6.731	.153	-3.845	23.514
Vocal Stimuli	8.801	4.963	.085	-1.284	18.887
Control Stimuli	4.148	4.900	.403	-5.809	14.106

TAP LOCATION

Stimulus Category	Mean Difference Classical – Jazz Participants	Std. Error	Sig.	95% Confidence Interval Lower Bound	95% Confidence Interval Upper Bound
Classical Stimuli	4.325	8.884	.630	-13.729	22.379
Jazz Stimuli	8.034	8.087	.328	-8.402	24.469
Vocal Stimuli	6.179	7.521	.417	-9.105	21.464
Control Stimuli	4.609	6.736	.498	-9.080	18.299

TAP VARIABILITY

Stimulus Category	Mean Difference Classical – Jazz Participants	Std. Error	Sig.	95% Confidence Interval Lower Bound	95% Confidence Interval Upper Bound
Classical Stimuli	5.591	2.049	**.010**	1.427	9.754
Jazz Stimuli	3.300	1.541	**.039**	.168	6.431
Vocal Stimuli	4.445	1.648	**.011**	1.096	7.794
Control Stimuli	4.243	1.691	**.017**	.807	7.680

Table A6. Results of models with fixed effects of Group, Stimulus Genre and Performance Mode and Random Intercept (Participant) in Experiment 2. P-center location and variability for Sung and Spoken Vocal stimuli.

CLICK LOCATION
(Full model AIC = 7679; Null model: Intercept + Random Intercept (Participant) AIC = 7746)

Fixed Effects						95% Confidence Interval	
Parameter	Estimate	Std. Error	df	t	Sig.	Lower Bound	Upper Bound
Intercept	-6.559	8.946	102.549	-.733	.465	-24.302	11.184
Group=Classical	22.244	10.777	51.495	2.064	.044	.612	43.875
Group=Jazz	0	0	.	.	.	.	.
Stimulus Genre=Classical	30.467	8.102	710.922	3.760	<.001	14.560	46.373
Stimulus Genre=Jazz	21.638	6.404	710.890	3.379	<.001	9.065	34.212
Stimulus Genre =Neutral	0	0	.	.	.	.	.
Performance Mode=Spoken	18.751	4.945	710.934	3.792	<.001	9.042	28.460
Performance Mode=Sung	0	0	.	.	.	.	.
Stimulus Genre=Classical* Performance Mode=Spoken	-21.764	6.999	710.952	-3.109	.002	-35.505	-8.022
Stimulus Genre=Classical * Performance Mode=Sung	0	0	.	.	.	.	.
Stimulus Genre=Jazz * Performance Mode=Spoken	0	0	.	.	.	.	.
Stimulus Genre=Jazz * Performance Mode=Sung	0	0	.	.	.	.	.
Stimulus Genre=Neutral * Performance Mode=Spoken	0	0	.	.	.	.	.
Group=Classical * Stimulus Genre=Classical	2.383	8.568	710.941	.278	.781	-14.438	19.205
Group=Classical * Stimulus Genre=Jazz	-1.238	8.565	710.938	-.145	.885	-18.053	15.577
Group=Classical * Stimulus Genre=Neutral	0	0	.	.	.	.	.
Group=Jazz * Stimulus Genre=Classical	0	0	.	.	.	.	.

Random Effects					95% Confidence Interval	
Parameter	Estimate	Std. Error	Wald Z	Sig.	Lower Bound	Upper Bound
Random Intercept (Participant) Variance	420.014	142.295	2.952	.003	216.215	815.906

CLICK VARIABILITY

(Full model AIC = 2269; Null model: Intercept + Random Intercept (Participant) AIC = 2319)

Fixed Effects						95% Confidence Interval	
Parameter	Estimate	Std. Error	df	t	Sig.	Lower Bound	Upper Bound
Intercept	14.714	7.827	110.422	1.880	.063	-.796	30.224
Group=Classical	21.221	9.243	57.536	2.296	.025	2.716	39.727
Group=Jazz	0	0	.	.	.	.	.
Stimulus Genre=Classical	14.717	7.383	219.000	1.993	.047	.166	29.268
Stimulus Genre=Jazz	5.297	5.853	219.000	.905	.367	-6.239	16.833
Stimulus Genre =Neutral	0	0	.	.	.	.	.
Performance Mode=Spoken	2.440	4.500	219.000	.542	.588	-6.428	11.308
Performance Mode=Sung	0	0	.	.	.	.	.
Stimulus Genre=Classical* Performance Mode=Spoken	-3.660	7.800	219.000	-.469	.639	-19.033	11.712
Stimulus Genre=Classical * Performance Mode=Sung	2.673	7.800	219.000	.343	.732	-12.699	18.045
Stimulus Genre=Jazz * Performance Mode=Spoken	0	0	.	.	.	.	.
Stimulus Genre=Jazz * Performance Mode=Sung	0	0	.	.	.	.	.
Stimulus Genre=Neutral * Performance Mode=Spoken	0	0	.	.	.	.	.
Group=Classical * Stimulus Genre=Classical	0	0	.	.	.	.	.
Group=Classical * Stimulus Genre=Jazz	-9.840	6.363	219.000	-1.546	.123	-22.381	2.701
Group=Classical * Stimulus Genre=Neutral	0	0	.	.	.	.	.
Group=Jazz * Stimulus Genre=Classical	0	0	.	.	.	.	.

Random Effects					95% Confidence Interval	
Parameter	Estimate	Std. Error	Wald Z	Sig.	Lower Bound	Upper Bound
Random Intercept (Participant) Variance	280.050	97.617	2.869	.004	141.427	554.547

Table A7. Pairwise comparisons of Stimulus Categories in Experiment 2. Significant results in **bold**. Bonferroni corrected for multiple comparisons.

(I) Stimulus Category	(J) Stimulus Category	Mean Difference (I-J)	Std. Error	Sig.	95% Confidence Interval Lower Bound	95% Confidence Interval Upper Bound
**CLICK LOCATION**						
Sung Classical	Sung Jazz	10.796	4.330	.203	-2.645	24.237
	Spoken Classical	2.067	4.676	1.000	-12.447	16.580
	Spoken Jazz	-8.829	6.393	1.000	-28.673	11.014
	Spoken Neutral	11.959	4.045	.071	-.597	24.514
Sung Jazz	Spoken Classical	-8.729	4.030	.409	-21.239	3.780
	Spoken Jazz	-19.626*	5.733	**.023**	-37.420	-1.831
	Spoken Neutral	1.162	4.268	1.000	-12.085	14.410
Spoken Classical	Spoken Jazz	-10.896	4.143	.150	-23.756	1.964
	Spoken Neutral	9.892	5.345	.771	-6.698	26.482
Spoken Jazz	Spoken Neutral	20.788*	5.874	**.018**	2.554	39.022
**CLICK VARIABILITY**						
Sung Classical	Sung Jazz	6.253	4.447	1.000	-7.550	20.057
	Spoken Classical	7.371	5.240	1.000	-8.894	23.636
	Spoken Jazz	3.862	6.599	1.000	-16.620	24.345
	Spoken Neutral	10.501	3.918	.134	-1.662	22.663
Sung Jazz	Spoken Classical	1.118	3.693	1.000	-10.346	12.581
	Spoken Jazz	-2.391	4.143	1.000	-15.251	10.469
	Spoken Neutral	4.248	2.622	1.000	-3.891	12.386
Spoken Classical	Spoken Jazz	-3.508	4.079	1.000	-16.169	9.152
	Spoken Neutral	3.130	3.010	1.000	-6.212	12.472
Spoken Jazz	Spoken Neutral	6.638	3.528	.726	-4.311	17.588

"Me" Sound, Classical Singer

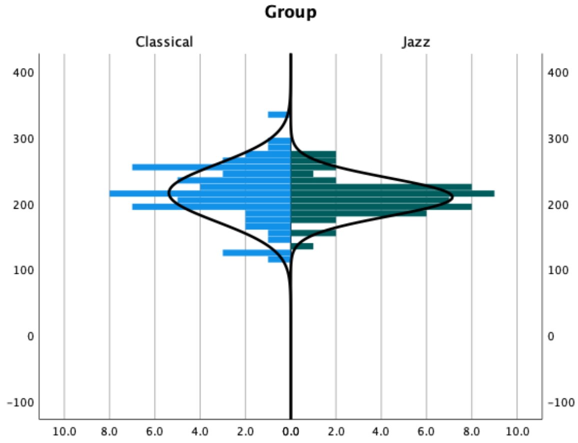

"Me" Sound, Jazz Singer

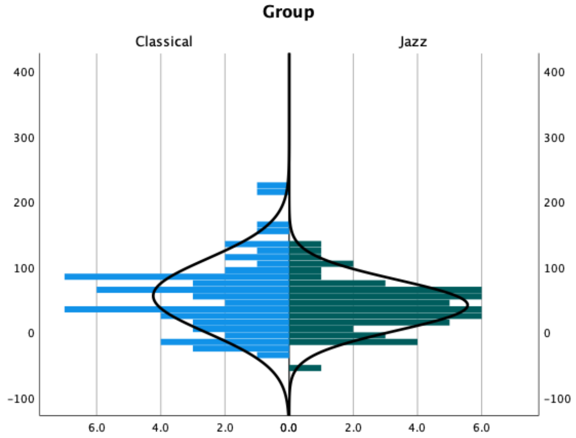

"Ma" Sound, Classical Singer

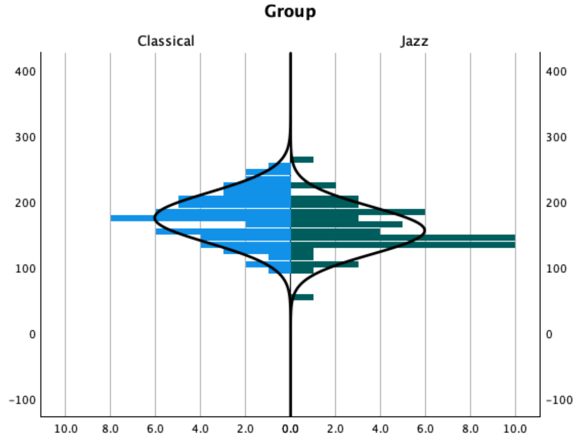

"Ma" Sound, Jazz Singer

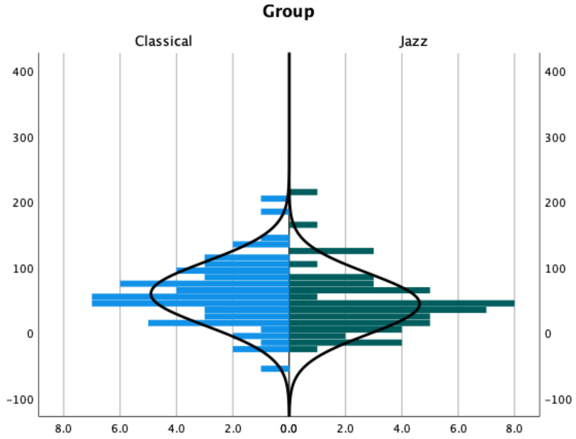

"E" Sound, Classical Singer

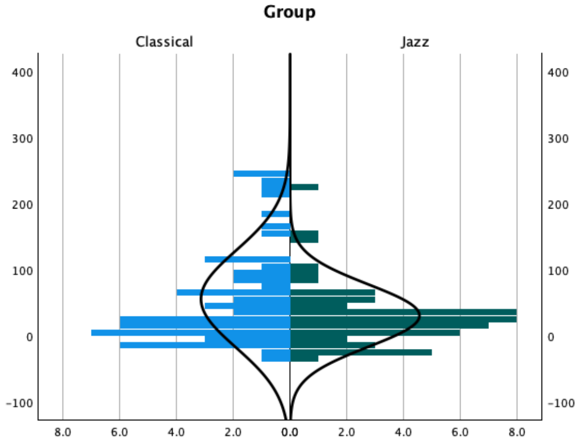

"E" Sound, Jazz Singer

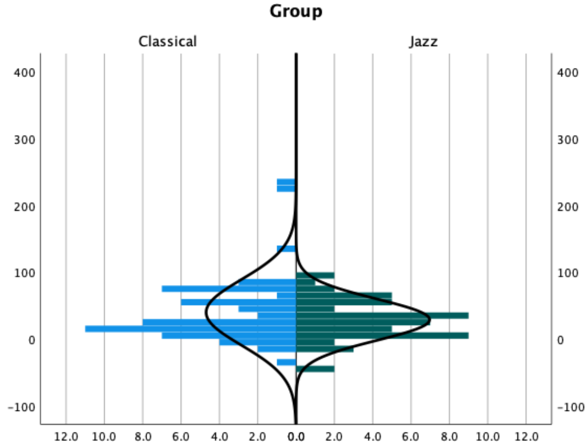

“A” Sound, Classical Singer

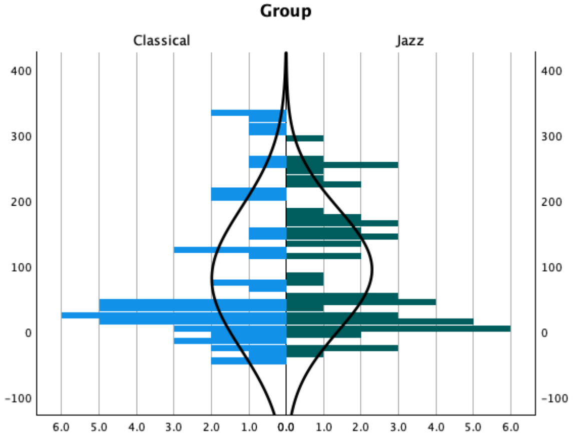

A” Sound, Jazz Singer

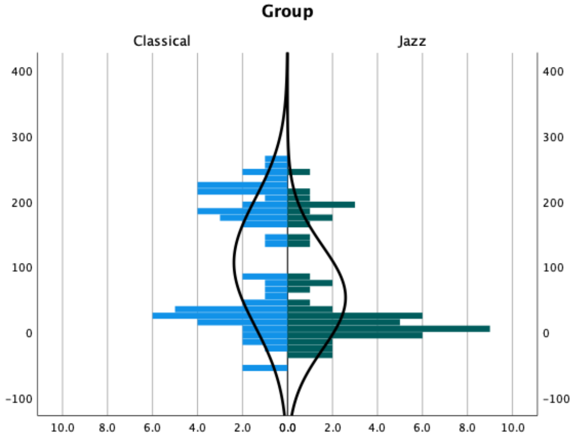

Figure A8: Pyramid histograms of responses for all vocal stimuli in the CLICK task, classical vs. jazz participant groups, fitted with normal distribution curves. X-axis = frequency, Y-axis = milliseconds.
